# Widespread distribution of supraglacial lakes around the margin of the East Antarctic Ice Sheet

**DOI:** 10.1038/s41598-019-50343-5

**Published:** 2019-09-25

**Authors:** Chris R. Stokes, Jack E. Sanderson, Bertie W. J. Miles, Stewart S. R. Jamieson, Amber A. Leeson

**Affiliations:** 1 Department of Geography, Durham University, Durham, DH1 3LE UK; 2 Lancaster Environment Centre/Data Science Institute, Lancaster University, Bailrigg, Lancaster LA1 4YW UK

**Keywords:** Cryospheric science, Climate change

## Abstract

Supraglacial lakes are important to ice sheet mass balance because their development and drainage has been linked to changes in ice flow velocity and ice shelf disintegration. However, little is known about their distribution on the world’s largest ice sheet in East Antarctica. Here, we use ~5 million km^2^ of high-resolution satellite imagery to identify >65,000 lakes (>1,300 km^2^) that formed around the peak of the melt season in January 2017. Lakes occur in most marginal areas where they typically develop at low elevations (<100 m) and on low surface slopes (<1°), but they can exist 500 km inland and at elevations >1500 m. We find that lakes often cluster a few kilometres down-ice from grounding lines and ~60% (>80% by area) develop on ice shelves, including some potentially vulnerable to collapse driven by lake-induced hydro-fracturing. This suggests that parts of the ice sheet may be highly sensitive to climate warming.

## Introduction

Supraglacial lakes (SGLs) form when meltwater ponds in depressions on the surface of a glacier or ice sheet^1^. They range in size from a just a few metres to tens of kilometres in area^1–4^ and they play an important role in an ice sheet’s mass balance. Firstly, they decrease the ice surface albedo and increase the absorption of incoming solar energy, thereby setting up a positive feedback that may further enhance melting^5–7^. Secondly, their rapid drainage has been implicated in the collapse of floating ice shelves^8–12^, which can then cause increased ice discharge from tributary outlet glaciers^13,14^. Thirdly, the drainage of SGLs to the bed of grounded ice in Greenland has been linked to transient speed-ups in ice velocity^15–19^. This process has yet to be observed in Antarctica, although it has been noted that future warming could increase the connectivity between surface and basal hydrological systems^20^.

To date, much of the research on SGLs has focused on the Greenland Ice Sheet (GrIS)^1–3,5–7,15–19,21–25^ and, to a lesser extent, ice shelves in the Antarctic Peninsula, particularly Larsen B Ice Shelf^8–10^. Until very recently, there were fewer reports of their existence in East Antarctica, which hosts the world’s largest ice sheet, and where surface melting is thought to be less intense and generally restricted to near-coastal regions^26–29^. However, the margins of the EAIS extend to latitudes of ~66° S (comparable to the Larsen B Ice Shelf), which contrasts with the margins of the neighboring West Antarctic Ice Sheet (typically south of 75° S). Moreover, several recent studies have shown that SGLs are more widespread than previously thought, and are often connected to channelized drainage systems on the ice sheet surface^4,20,30–33^. Indeed, it has been noted that future warming could increase surface melt and that such drainage systems could deliver excess meltwater to ice shelves that may be vulnerable to collapse^4,11,20,29,34^, unless surface channels export meltwater off the ice shelf^33^.

Despite recent progress, the underlying processes governing meltwater production, ponding and runoff in East Antarctica remain unclear^20,33^. For example, Antarctic-wide estimates of surface meltwater production are now available from both satellite data and regional climate modelling^20,28^, but there are, as yet, no comprehensive and consistent observations of where excess meltwater is ponding and generating SGLs across the entire ice sheet surface. One recent study^4^ identified ~700 drainage systems (SGLs connected to surface streams), but this was thought to be an underestimation due to the spatial and temporal coverage of the satellite imagery, which was acquired from multiple years and not necessarily from the summer melt season. A recent review^20^ of Antarctic surface hydrology highlighted the need for robust observations of processes related to surface melt, noting that new high-resolution satellite sensors (such as Landsat 8 and Sentinel) could help address this data void.

In this paper, we analyze ~5 million km^2^ of imagery from Landsat 8 and Sentinel 2A and use a consistent semi-automated approach to identify and quantify the distribution and extent of SGLs produced around the peak of a single melt season in January 2017 (see Methods). Note that the austral summer of 2016/2017 was characterised by an unprecedented retreat of Antarctic sea ice linked to atmospheric circulation anomalies^35^, which may also have increased ice sheet surface melting. With this in mind, our aim is to determine where meltwater is ponding in SGLs and to use these spatial patterns to understand the controls on their distribution. We deliberately focus on capturing the first consistent observations of their spatial distribution across the whole ice sheet, rather than tracking their temporal evolution through time, which is perhaps better suited to regional studies^30,31^ and which is currently precluded by image availability across the whole ice sheet. We then discuss their potential impact on ice sheet mass balance and stability, specifically in relation to ice shelves that are deemed potentially vulnerable to hydro-fracturing^11^.

## Results

The duration of the East Antarctic melt season varies according to location and from year to year, but mid- to late-January typically coincides with the warmest part of the austral summer in most marginal regions of the ice sheet^26,27,31,36^. Given our focus on ascertaining a consistent record of the spatial extent of SGLs around the peak of the melt season, we therefore limited our imagery to January 2017. This is a part of the first Antarctic (austral) summer to benefit from the near-complete coverage of medium-resolution imagery from the Sentinel 2A (up to 10 m) and Landsat-8 (up to 15 m) satellites. Due to the difficulty of acquiring a single image mosaic of the whole ice sheet on the same day (or even from within a period of a few weeks), these images span 1^st^ January to 31^st^ January 2017, but with the majority from mid- to late January (see Supplementary Figs 1 and 2).

**Figure 1:**
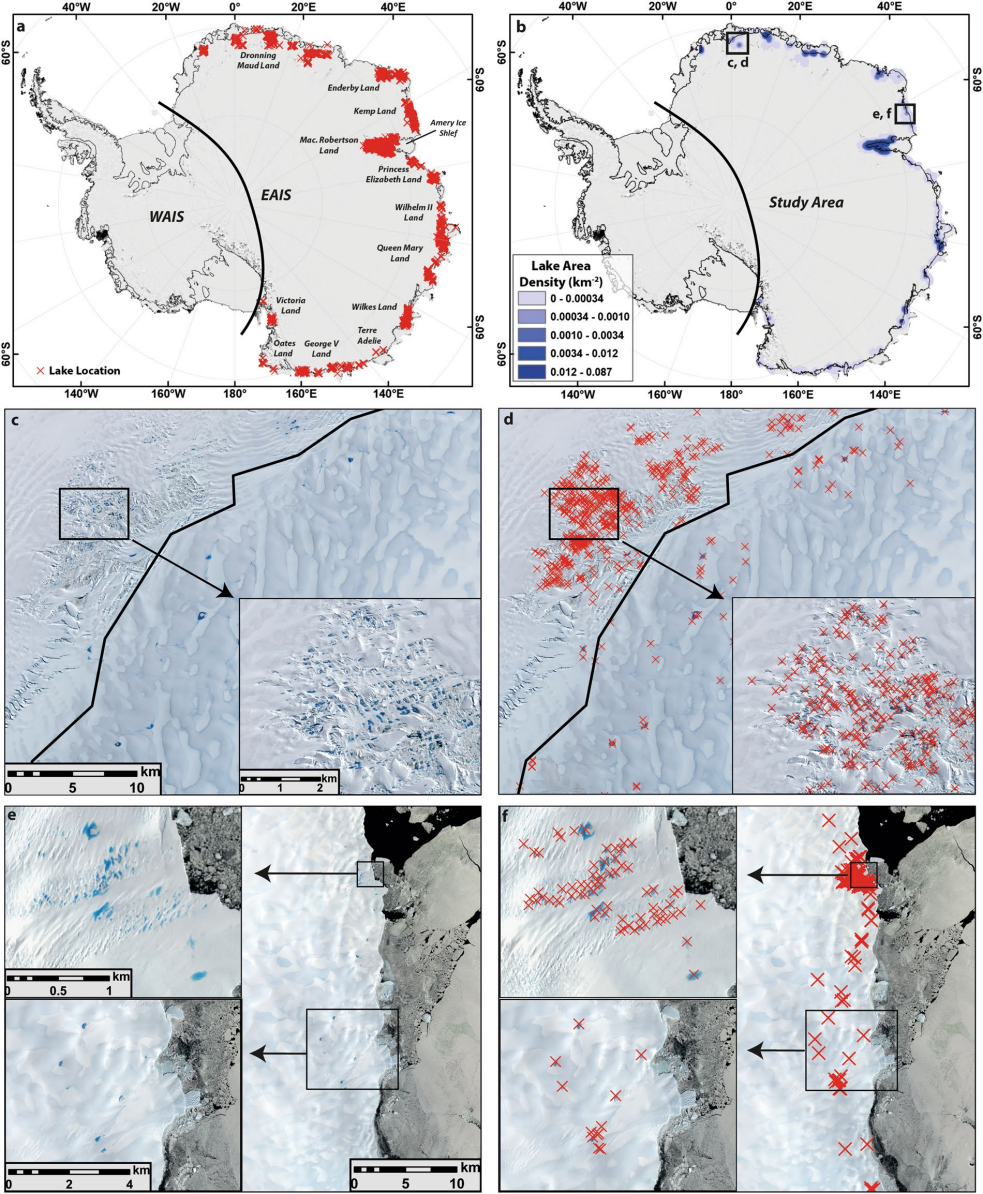
Location and density of supraglacial lakes (SGLs) in East Antarctica, alongside examples. (**a**) Location of 65,459 mapped lakes that appeared on imagery from January 2017, each marked by a red cross. (**b**) Lake density map showing the cumulative area of SGLs within 1 km^2^ cells using a 50 km search radius. (**c**,**d**) Sentinel 2A satellite image (12^th^ Jan 2017) of the high density of lakes on the Jutulstraumen Glacier, Dronning Maud Land. Note that lakes have developed above and beyond the grounding line (thick black line), but there is a clustering of lakes 5–10 km down-ice from the grounding line. (**e**,**f**) Sentinel 2A satellite image (27^th^ Jan 2017) of clusters of lakes towards the ice sheet margin in Kemp Land.

Our dataset includes 65,459 SGLs that had formed on the EAIS in January 2017 (digital shapefiles (.shp) available in Supplementary Data 1). We find that SGLs are far more widespread than previously recognized and, similar to Greenland, occur in most peripheral regions of the ice sheet with particularly high densities in Wilkes Land, Queen Mary Land, Mac. Robertson Land, Enderby Land and Dronning Maud Land (Fig. 1). Significantly, lake area densities in these regions, including on both grounded and floating ice, are ~0.05 km^−2^, which is similar to mean values (0.07 km^−2^) reported for well-studied regions of the GrIS^3^. We also discover SGLs in several regions where their widespread development has not been previously reported, such as Kemp Land, Terre Adélie and George V Land (Fig. 1).

**Figure 2:**
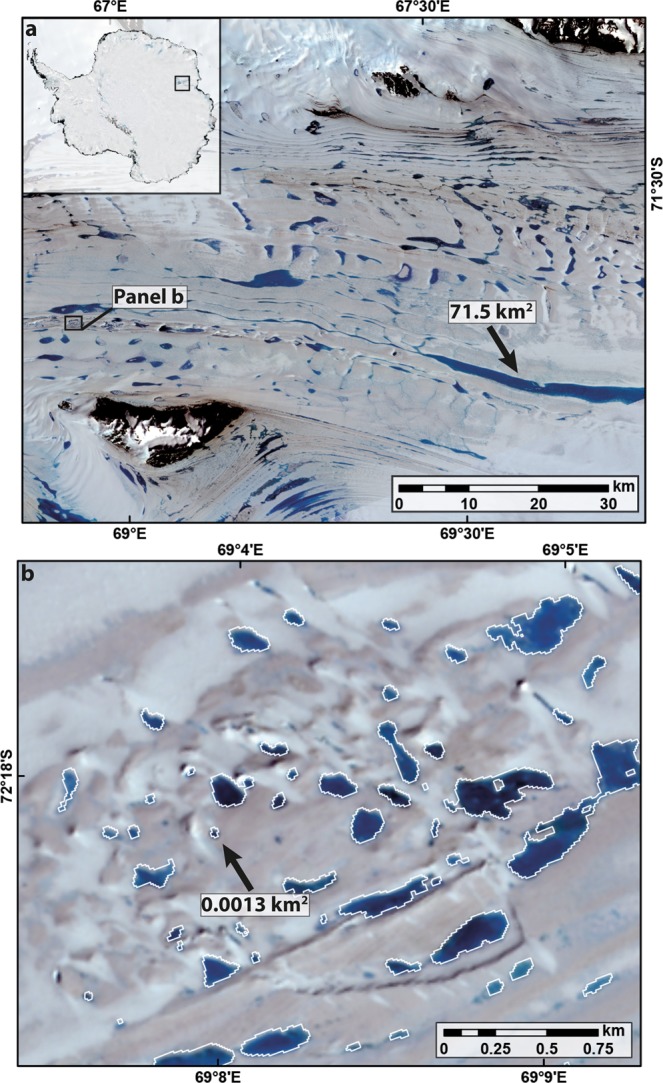
Examples of the high density and range of lake sizes on the the Amery Ice Shelf, East Antarctica (see also Fig. 1). This includes the largest lake in the dataset (**a**) and examples of small lakes (**b**) that are close to the median lake size in our dataset.

**Figure 3:**
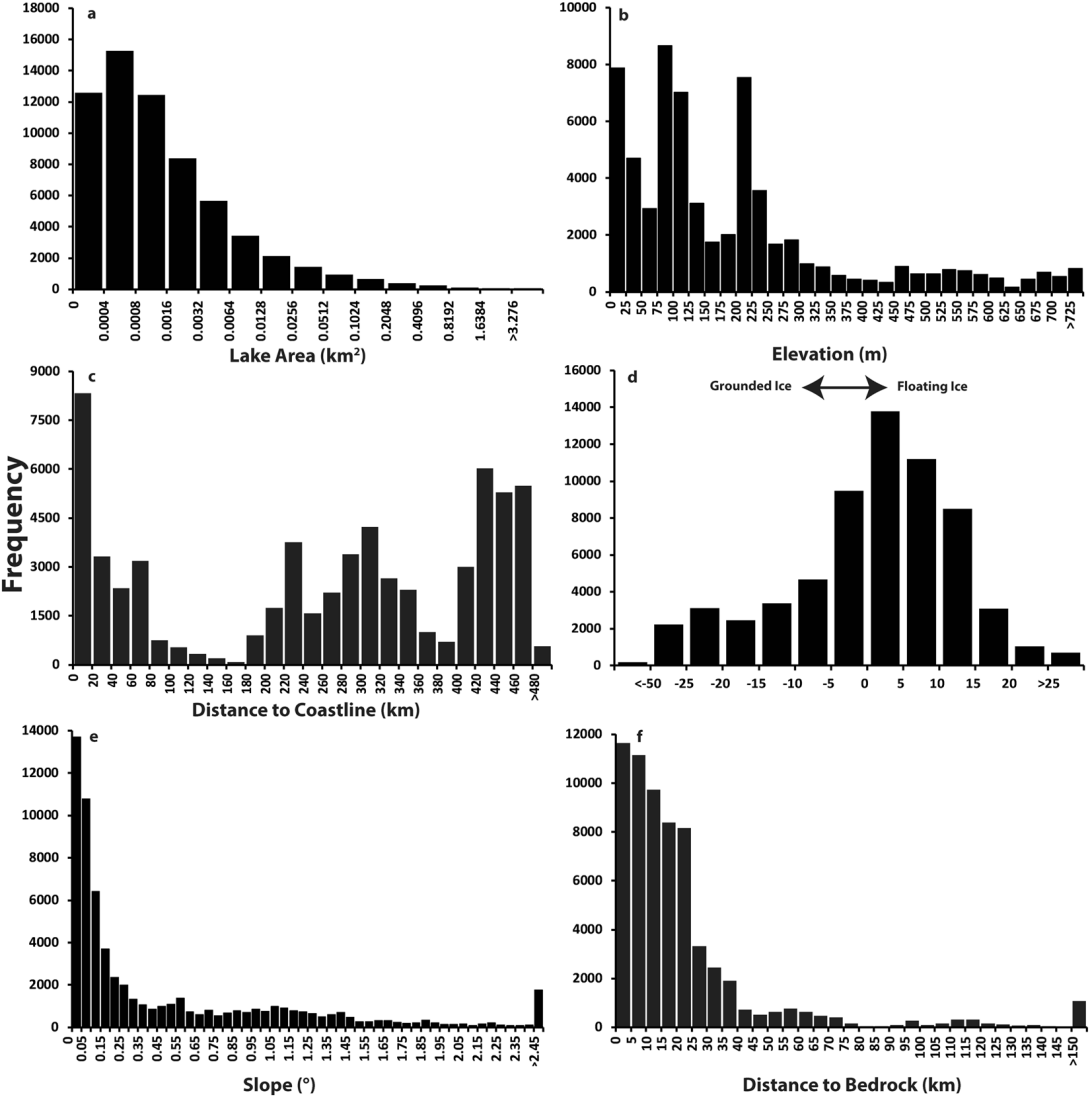
Frequency distributions of supraglacial lakes in East Antarctic by topographic variables. (**a**) individual lake areas (note that bin intervals increase by 50% from left to right); (**b**) individual lake elevations; (**c**) distance of each lake to the ice margin/coastline; (**d**) distance of each lake to the grounding line (negative values indicate up-ice from the grounding line); (**e**) ice surface slope; (**f**) distance of each lake to nearest exposed bedrock.

The cumulative area of SGLs amounts to 1,383.5 km^2^ (±13.8 km^2^) and individual lakes range in area from 0.0002 km^2^ (our minimum threshold for lake detection: see Methods) to a maximum of 71.5 km^2^ (±0.7 km^2^). It is likely that our analysis misses very small lakes that fall below our minimum threshold, but they are unlikely to add substantially to the total area (see Methods). The largest lake is an elongate feature tens of kilometres long on the Amery Ice Shelf, Mac. Robertson Land (Fig. 2a). Previous work noted a lake in this location, which likely forms seasonally in a longitudinal trough in the ice surface^4,37^. It is far larger than some of the largest lakes reported from the grounded portions of the GrIS (e.g. 16.9 km^2^, ref.^2^), but lakes approaching this size are rare: only 11 lakes in our dataset from East Antarctica are >10 km^2^. Indeed, the size-frequency distribution of SGLs in East Antarctica is highly skewed towards smaller lakes (Fig. 3a), with a mean of 0.021 km^2^ and a median of 0.001 km^2^ (st. dev. 0.459 km^2^). Similar positively-skewed size-frequency distributions (albeit with much smaller sample sizes) have been reported from the GrIS^3,21–23,25^ and the Antarctic Peninsula^3,9^ (Supplementary Fig. 7). Our mean and median values are, however, much smaller than most other inventories, due to our semi-automated method and its application to much higher resolution imagery across the entire ice sheet^23,25^.

**Figure 4:**
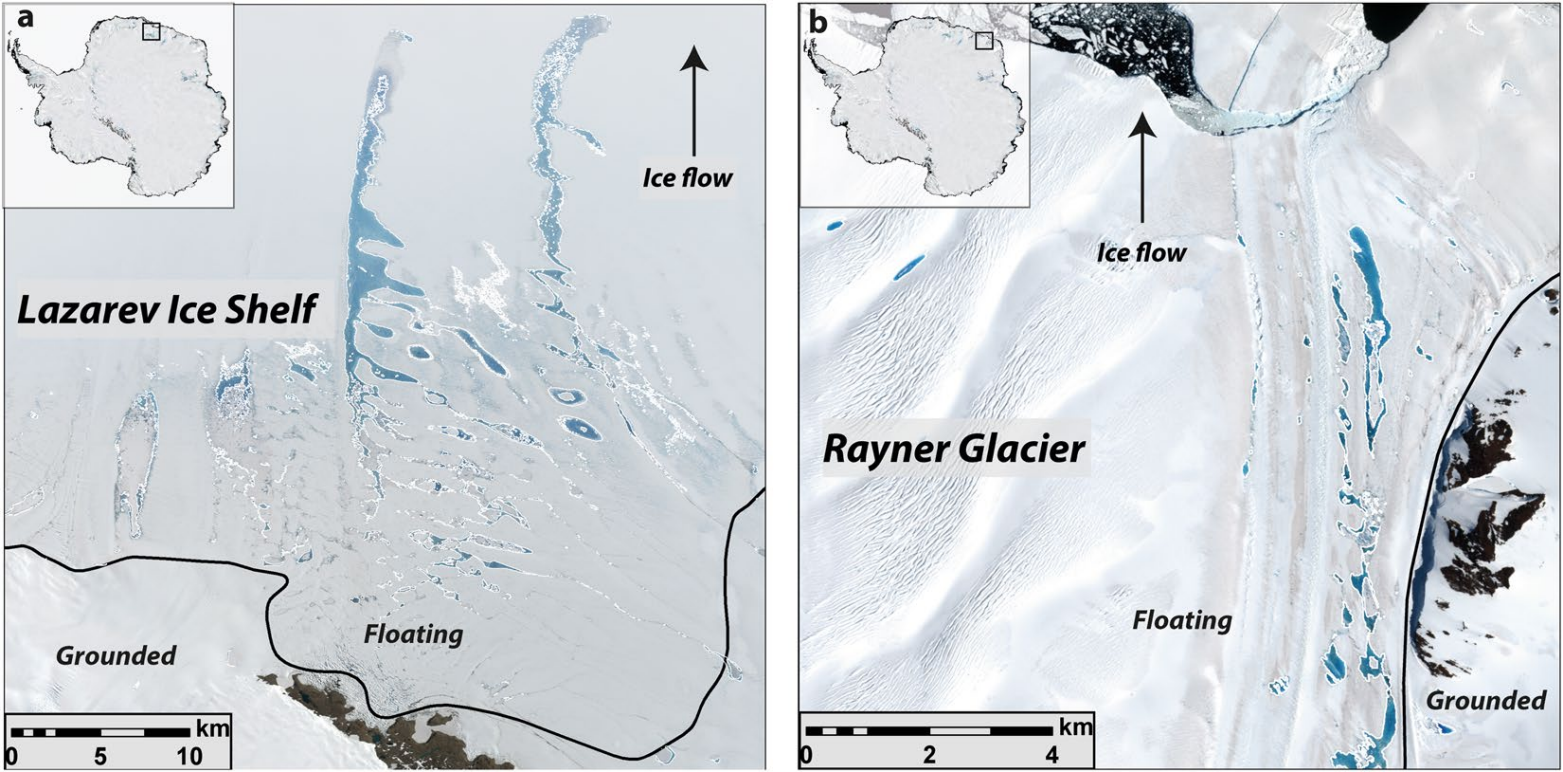
Example of lakes clustered a few kilometres down-ice of grounding line locations (black line). (**a**) Large lakes forming close to the grounding line on the Lazarev Ice Shelf in Dronning Maud Land, and which appear to drain into the snow-pack/firn. (**b**) An example of lakes clustered near the lateral margin of the Rayner Glacier, near to nunataks in Enderby Land. In both locations, SGLs are advected with ice flow.

The potential influence of SGLs on ice sheet dynamics will depend on whether they form on grounded ice or floating ice shelves^4,20^. Using a previously-published grounding line map^38^, we find that 38,476 lakes (60.4% of lakes, but comprising 81.6% of the total lake area) occur on floating ice, with a clear clustering of lakes within a few kilometres down-ice from grounding line locations (Figs 3d and 4). Indeed, SGLs do not gradually increase in number towards the margin of the ice sheet, most of which is floating. Rather, there are numerous instances where lake occurrence decreases and they are absent towards the calving front of ice shelves (e.g. Fig. 4a), which has been observed in other localized studies^30,32^. An analysis of the distance of each lake to the ice margin indicates a modal value (13%) between 0 (representing the ice margin) and 20 km from the margin (Fig. 3c), but the distribution is clearly multimodal, with other peaks occurring much further inland. This perhaps seems counterintuitive, but it is due to large clusters of SGLs that exist at low elevations and below (down-ice from) the grounding line on the Amery Ice Shelf (Figs 1 and 2), which extends >500 km inland from the ice sheet margin. More generally, ~38% of lakes in East Antarctica occur at elevations from 0 to 100 m (Fig. 3b) and the vast majority of lakes (80.6%) form on slopes <1° (Fig. 3e). This clearly relates to their prevalence on low-elevation ice shelves with low surface slopes.

**Figure 5:**
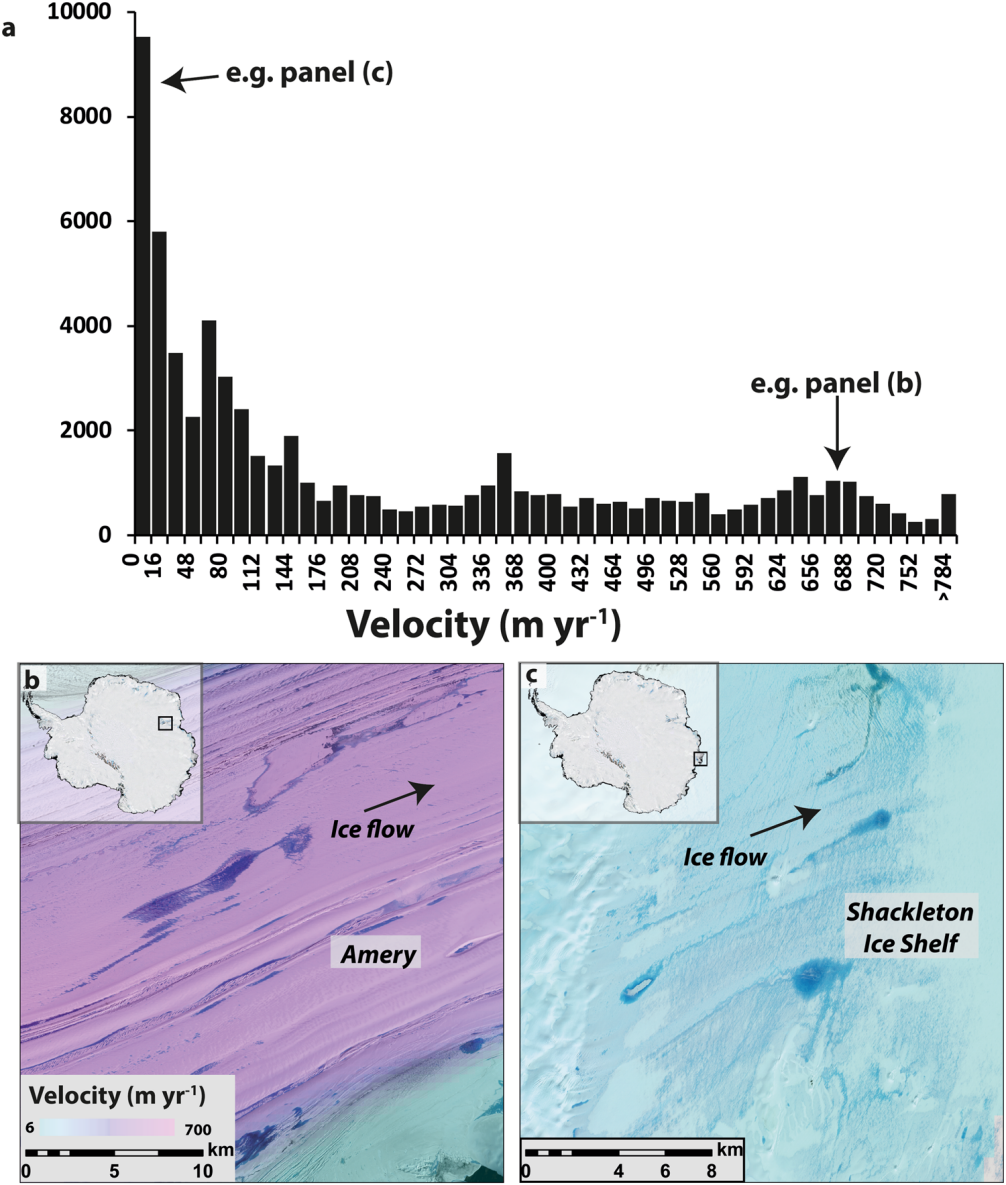
Frequency distribution of supraglacial lakes related to ice velocity, with examples on fast- and slow-flowing ice. (**a**) Frequency distribution indicating that lakes tend to form on slow-moving ice, but exist on a wide range of ice velocities. (**b**) Example of lakes on fast-flowing ice (~700 m a^−1^) on the Amery Ice Shelf, which tend to form elongate ponds in between longitudinal flow-stripes. (**c**) Example of lakes flowing on slow-flowing ice on the Shackleton Ice Shelf.

There is also a positive skew when the frequency distribution of lakes is plotted by ice velocity (Fig. 5), which also reflects the preferential development of lakes on slower-flowing ice shelves with low surface slopes. A similar analysis^4^ from a much smaller sample in Antarctica (n = 700) found two thirds of surface drainage features (streams and ponds) originated on ice flowing <120 m a^−1^. For comparison, we find that just less than half of the population of lakes (48.9%) formed on ice flowing <120 m a^−1^. Indeed, thousands of lakes form on rapidly-flowing ice (i.e. >500 m a^−1^) and these tend to form in longitudinal troughs between flow-stripes on outlet glaciers and/or where they transition into ice shelves (e.g. Figs 2a, 4b and 5b). Large numbers of lakes in the upper reaches of the Amery Ice Shelf are likely to account for some of the peaks in lake frequency on ice flowing at higher velocities (Fig. 5a,b).

**Figure 6:**
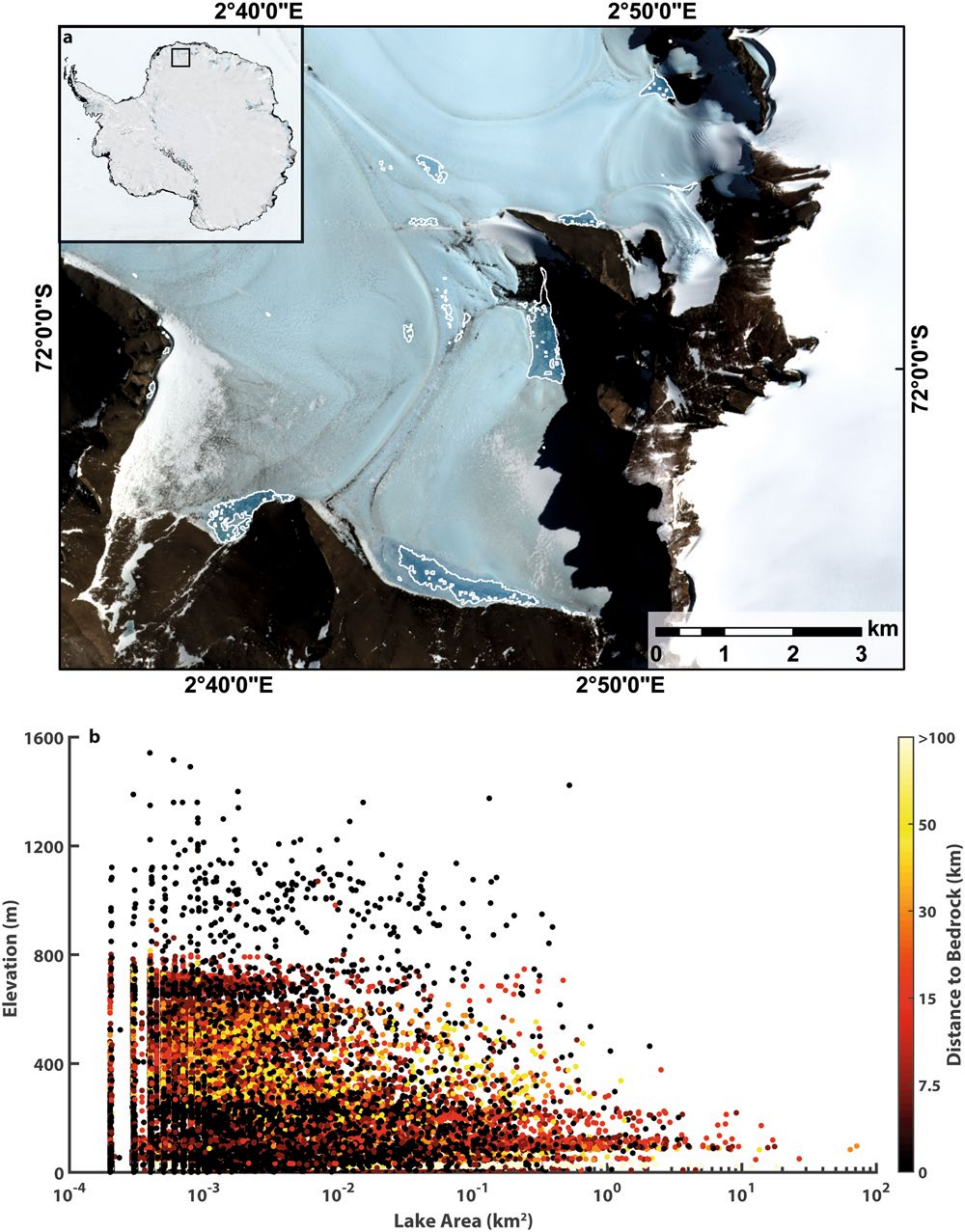
Influence of exposed bedrock on supraglacial lake development at high elevations. (**a**) Sentinel 2A satellite image (12^th^ Jan 2017) of SGLs (white outlines) at high elevations (>1000 m) close to nunataks in the upper catchment of Jutulstraumen Ice Stream, Dronning Maud Land. (**b**) Scatterplot of lake area versus lake elevation colored by distance to exposed rock. Note that the vast majority of lakes at the highest elevations (e.g. >800 m) exist in close proximity (<7.5 km) to exposed bedrock.

Despite their predominance at low elevations (Fig. 3b) and below (down-ice from) grounding line locations (Fig. 3d), we also find that SGLs extend to much higher elevations and further inland than previously recognized in East Antarctica. The number of lakes above the grounding line (25,267) represents ~40% of the total population and their cumulative area (253 ± 2.53 km^2^) equates to ~18% of the total. Thousands of lakes exist at elevations >500 m and hundreds >1000 m (Figs 3b and 6). These elevations are higher than reported in most previous localized studies in East Antarctica, which generally found lakes up to only a few hundred metres in elevation^30,31,36^, although small surface streams have been observed up to 1,830 m in the Transantarctic Mountains^20^. Thus, our observations reveal upper elevations (1583 m: Fig. 6) that are close to those observed on the GrIS^21,24^. Where lakes form far inland and at higher elevations, it has been noted that they are typically associated with nunataks^4^. We find that 35% of SGLs form within 10 km of exposed bedrock (Fig. 3f) and, at the highest elevations (>800 m), ~90% of lakes occur within 2 km of exposed bedrock (Fig. 6b).

## Discussion

### Controls on the occurrence and distribution of SGLs

At the ice-sheet scale, our observations reveal that the development of SGLs is clearly influenced by ice surface slope (Fig. 3e) and that the majority of lakes form at low elevations (<300 m: Fig. 3b) close to and just beyond the grounding line (Figs 3d and 4). The link between ice surface elevation and slope is unsurprising, given that low elevation areas are typically warmer and low surface slopes are conducive to the ponding of excess meltwater; similar relationships have been observed on the GrIS^21^. The clustering of lakes near to and immediately down-ice from grounding lines is, perhaps, less intuitive, but recent work has shown that this can be explained by regional scale wind-patterns and localized ice-albedo effects^20,32^. Low elevation areas close to the grounding line in East Antarctica are often subject to persistent katabatic winds from the ice sheet interior^20,32^. These winds warm and mix the air as it flows downwards, leading to near-surface summer temperatures that can be >3 °C higher than regions further up- or down-ice, and with the consequence that meltwater production is doubled close to the grounding line compared to areas farther down-ice^32^. Moreover, strong near-surface winds result in substantial snow erosion, exposing lower-albedo blue-ice areas near grounding lines that further enhance surface melting in those regions^32,39^.

Surface drainage systems can also deliver water from higher elevation grounded ice to lower elevation areas, whereupon it is more likely to be stored on flatter surfaces just beyond the grounding line^4,31,32^. In some locations, these channels can transport meltwater across the ice shelf and directly into the ocean^33^, but it is far more common for the channels to drain into new lakes or simply terminate, beyond which SGLs are generally absent^30,32^ (Figs 4a and 5c). Their absence towards the outer limits of ice shelves is, again, less intuitive, but likely reflects the lower melt-to-accumulation ratios in near coastal regions^32^. That is, although melt duration and intensity generally increase towards the coast^27,28,40^, accumulation (snowfall) is also higher and the firn air content (FAC) is correspondingly higher^32^. This allows any surface melt to percolate into the firn and precludes the formation of SGLs. However, whilst it was generally assumed that most East Antarctic ice shelves had a high FAC, recent evidence has shown that this may not be the case and that extensive meltwater-induced firn air depletion is occurring^32^. This is because SGLs and channels down-stream of grounding lines are draining to englacial positions, and then being advected within the ice shelf and progressively submerged beneath snow/firn^32^. Thus, large areas of saturated firn and/or refrozen ice lenses may exist in several East Antarctic ice shelves and are likely to influence the vulnerability of ice shelves to hydrofracturing^11,41^ (discussed below).

Elsewhere, blue-ice areas and the presence of nunataks would appear to be the most important controls on the far more sporadic appearance of SGLs at higher elevations^4,20,36^ (Fig. 6). Exposed bedrock and blue-ice areas have a lower albedo and locally increase the absorption of incoming solar energy^32,36,39^. Moreover, nunataks and blue-ice areas tend to co-exist because exposed bedrock not only increases melt through its lower albedo, but may also locally increase wind speeds, thereby further lowering the ice/snow surface and increasing the area of exposed rock and blue-ice^4^. As noted previously^4^, these effects are likely to be particularly important in explaining the development of lakes at high elevations (see Fig. 6) that would otherwise be too cold for surface melting and which can be difficult to capture in regional climate models^42^.

On a more local scale, a precondition for the development of SGLs are ice surface depressions and our analysis suggest that these are available across a wide range of ice velocities (Fig. 5). Large numbers of lakes form on slow-flowing ice, with around half occurring on ice flowing <120 m a^−1^. This likely reflects the large number of lakes on generally slow-flowing ice shelves, where surface topography is generated by a range of processes, such as surface flow-stripes and rumples inherited from grounded ice flow upstream^3^ or from internal stresses generated within ice shelves^3,43^. More recently, it has been found that basal channels formed by meltwater eroding the underside of ice shelves can also influence ice shelf surface topography and divert meltwater into surface fractures^44,45^. The filling and draining of fractures can also induce further ice shelf flexure and crevasse formation^10,12^. In contrast, surface depressions on grounded ice are closely linked to the underlying bed topography and the transmission of basal variability has been shown to increase under higher ice velocities^46^. Ice surface depressions related to underlying bedrock are likely to persist and are known to anchor lakes in the same location each year^1,3^. In other locations, longitudinal surface structures (known as ‘flow-stripes’) develop as a result of increased strain, particularly in areas of flow acceleration and confluence^47,48^. Large numbers of highly elongate SGLs are often found in the intervening troughs between flow-stripes (Figs 2a and 5b) and similar troughs can also form at the lateral shear margins of outlet glaciers/ice shelves^33^, where meltwater production might also be enhanced by the lower albedo of adjacent rock-walls (e.g. Fig. 4b).

In summary, SGLs occur in most low elevation, gently-sloping marginal areas of the EAIS, which reflects the fact that the ice sheet margin extends to relatively low latitudes where summer temperatures are high enough for surface melting to occur^20,27,28,40^. However, the distribution of lakes is highly variable and they occur in clusters of higher density (Fig. 1) that do not obviously correlate with the areas of highest melt obtained from radar backscatter^27,28^ or coarse-resolution (e.g. 27 km) regional climate modelling^40,42^. Rather, clusters of high lake density reflect the interaction between local-scale climatic controls and ice surface characteristics, including regional-scale wind patterns, ice surface albedo and topography, and firn air content and thickness^32,42,49^. Thus, the complex interplay of these local-scale processes makes it difficult to predict the location of SGLs based only on the current generation of Antarctic-wide observations and modelling of surface meltwater production.

### Potential impact on ice sheet mass balance

SGLs can potentially influence ice sheet mass balance in three main ways^20^, which we now consider: (i) reducing ice surface albedo and increasing ablation; (ii) draining to the ice sheet bed and locally enhancing basal lubrication; and (iii) draining through the ice and fracturing ice shelves, leading to their collapse.

The lower relative albedo of SGLs means that the ablation rate at the bottom of a lake can be up to two times higher than on a nearby ice surface^6^. Thus, the high density of SGLs in some regions of East Antarctica (Fig. 1) has the potential to locally enhance melt rates, which could create a more negative surface mass balance than would otherwise be the case. However, many of the lakes we observe, particularly those at higher elevations, are likely to re-freeze^27,40,50^, and others might simply drain into the firn layer, as can be observed on several ice shelves^30,32^ (e.g. Figs 4 and 5). Thus, unless the excess melt generated by SGLs is exported via surface channels and off the ice sheet^33^, this process is unlikely to impact on net surface mass balance^50^. It has been noted, however, that percolation and refreezing of meltwater into the firn layer can exert a localised warming effect on ice temperatures through the release of latent heat^51,52^. Indeed, recent work on one outlet glacier in East Antarctica has hypothesized that englacial penetration of meltwater and/or the filling of crevasses can increase the ice temperature, soften the ice, and lead to a seasonal speed-up of ice shelf flow^53^. Moreover, future warming is likely to increase surface melting in Antarctica^4,20,29,34,54,55^. Over long time-scales, this increase in melting could trigger a positive feedback whereby enhanced surface melt leads to surface lowering and a larger proportion of the ice sheet surface below the equilibrium line that is then subject to more intense melting^20^. Recent work from Greenland has demonstrated a clear correlation between total lake area and annual surface runoff^21^, as well as their inland expansion during more intense melt years and over decadal time-scales^7,22,24^. Localised studies in East Antarctic have found similar relationships^31,53^. Thus, any future warming in East Antarctica is likely to lead to similar increases in the cumulative area of SGLs and the locally-enhanced melt associated with their development is likely to play a greater role in the surface mass balance^20^.

The drainage of SGLs to the bed of the GrIS has been linked to transient speed-ups in the velocity of grounded ice^15–19^ but this process has yet to be observed in Antarctica^20^. However, we note that drainage events have been documented on floating ice in East Antarctica^31,32^ and our results provide a clear indication of where SGLs occur in the highest densities (Fig. 1). Furthermore, we estimate that hundreds of lakes in our dataset are likely to be of sufficient volume to drain, particularly those on floating ice. This is based on an area-volume scaling relationship derived from a linear regression (R^2^ = 0.86) of lake areas and volumes measured in two previous studies in Antarctica on Larsen B Ice Shelf^56^ and on the Langhovde Glacier, East Antarctica^31^. Similarly strong relationships have been found for lake areas and volumes on the GrIS^22,25,57^ and our approximation gives a total volume of ~1 km^3^ (~1 Gt liquid water), with maximum lake volumes up to 0.05 km^3^. Mean volumes are only 0.000015 km^3^ (st. dev. 0.0003 km^3^), but ~1,500 SGLs in our dataset (70% of these are on floating ice) are likely to contain water volumes that are greater than the average volume calculated for lakes on the Larsen B Ice Shelf prior to its collapse^56^ (Supplementary Fig. 7). Clearly, these estimates are only first order approximations and further work could use more sophisticated depth retrieval algorithms^25,58–60^ to explore the temporal evolution of lake volumes and to search for possible drainage events on both grounded and floating ice.

Potentially of most significance to ice sheet mass balance are the large number of lakes (~39,000) that we observe on floating ice shelves that fringe the EAIS. These lakes constitute 60.4% of the total number of lakes, but 81.6% of the total lake area. Their potential importance stems from observations that SGLs provide effective reservoirs to drive the process of hydro-fracturing, whereby meltwater fills crevasses and the resultant water pressure propagates the tip of the crevasse until full-thickness fracture occurs^8,61^. The filling and draining of SGLs has also been shown to induce ice-shelf flexing, which can also generate fractures^10,12,62^. Of particular concern is that many ice shelves exert a buttressing effect that restrains the flow of ice further upstream^63,64^. In such cases, their disintegration (e.g. via hydrofracturing), can trigger an acceleration in the flow velocity and discharge of their tributary outlet glaciers, as has been observed in the Antarctic Peninsula^13,14^.

Of particular relevance to our study is recent work that has shown that several ice shelves in East Antarctica are dynamically important in terms of buttressing inland ice^64^ (e.g. in Wilkes Land); and that some of these ice shelves may be more vulnerable to hydro-fracturing than previously thought^11^. Specifically, a recent study^11^ used the relationship between active microwave backscatter and average annual melt days to create a relative index that shows ice shelf vulnerability to surface-melt induced collapse via hydrofracture across Antarctica. Consistent with observations^41,52^, they found several ice shelves on the Antarctic Peninsula have significantly ice-saturated firn layers that make them vulnerable to surface meltwater ponding and hydrofracture^11^, but they also noted several ice shelves in East Antarctica had relatively high vulnerability indices, e.g. the Amery, West, and Shackleton ice shelves. As mentioned earlier, these ice shelves experience high melt rates due to localised katabatic winds converging over their grounding lines^20,32^. Shackleton Ice Shelf, for example, experiences upward of 60 days of melting per year^27^, with melt rates as high as 200 mm w.e. a^−1^ (ref.^28^).

**Figure 7:**
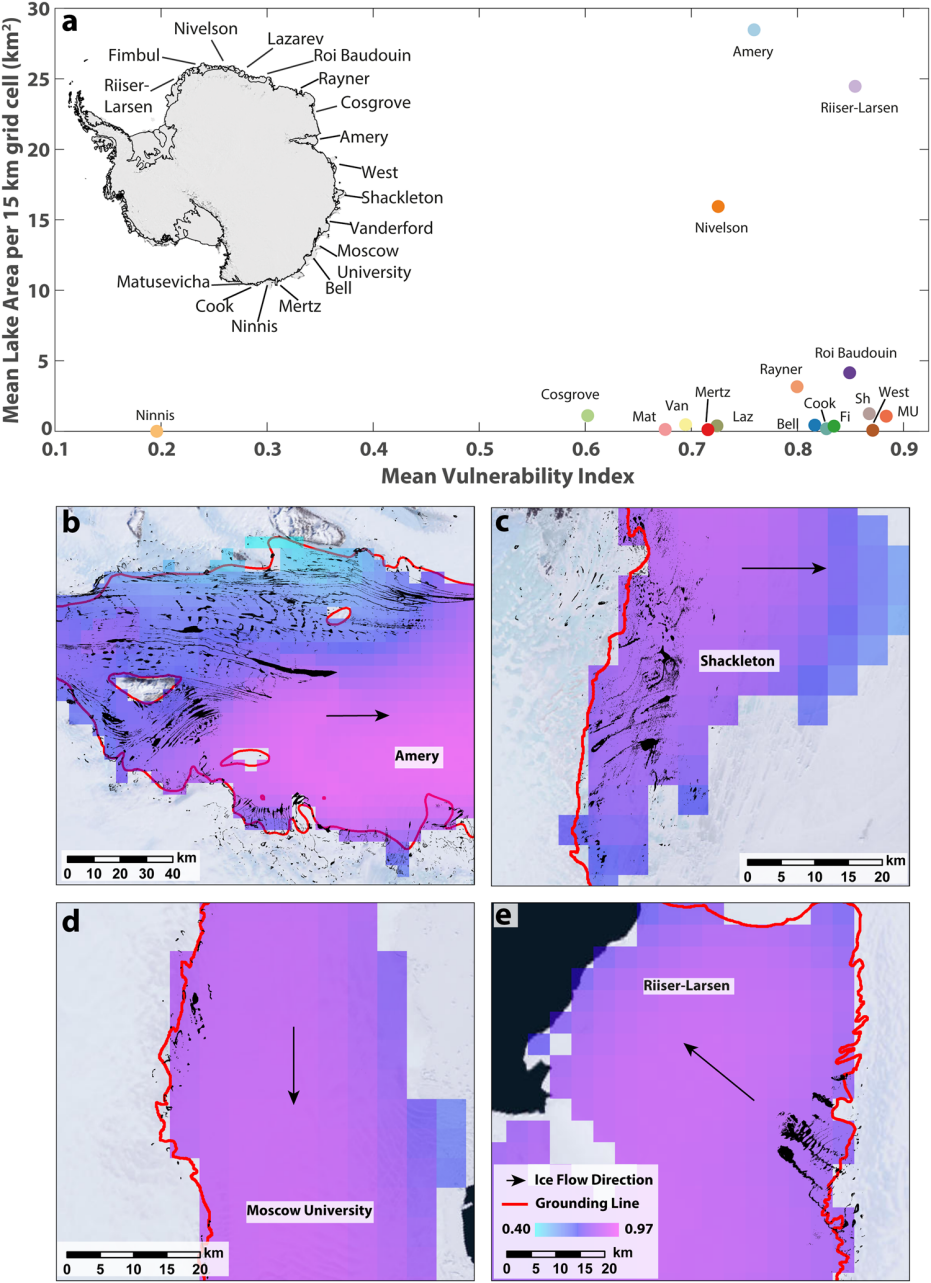
Supraglacial lake densities in relation to ice shelf vulnerability to hydrofracturing (from ref.^11^). (**a**) Scatterplot of mean ice shelf vulnerability (in regions where lakes are present) versus mean lake area per 15 km grid cell. Supraglacial lake locations (black polygons) overlain on ice shelf vulnerability index for four different ice shelves with high vulnerability indices, namely the (**b**) Amery, (**c**) Shackleton, (**d**) Moscow University, and (**e**) Riiser-Larsen. Grounding line locations shown in red line with ice flow direction approximated with back arrow.

Ice shelves with low FAC and high vulnerabilities were interpreted to have the potential to support SGLs^11^, but the presence/absence of lakes was not investigated in any detail. However, our data clearly show that high densities of lakes exist on some of the ice shelves deemed vulnerable to hydrofracturing, particularly near to the grounding lines of Amery, Shackleton and Moscow University ice shelves (Fig. 7b–d). We would, of course, expect to find lakes on ice shelves that are inferred to have ice-saturated firn layers, but our analysis provides a useful indication of where lakes are already developing in their highest densities, and also shows that some ice shelves with a high vulnerability index have very few lakes present (Fig. 7). Of particular concern, however, would be ice shelves which have a high vulnerability to fracturing, a high areal density of lakes, and which have been shown to be dynamically important in terms of their buttressing effect^64^. Moscow University Ice Shelf and Shackleton Ice Shelf would fit this category (Fig. 7) and the future evolution of SGLs in Wilkes Land is particularly important given future predictions of decreased FAC in this region^55^ and recent concerns over the retreat and mass loss from some of its outlet glaciers^65–67^. In contrast, although the Amery Ice Shelf supports the highest densities of lakes in our analysis and has a high vulnerability index^11^, its thickness and geometry within a narrowing embayment and several pinning points would suggest that it is highly unlikely to collapse^68^. This illustrates a very important point, which is that lake drainage, either on grounded or floating ice, will only occur when the necessary stress conditions are met^69^. Thus, hydrofracturing of ice shelves is not simply related to the distribution and volume of water in SGLs, but some stress condition, linked to ice shelf geometry and lateral boundary conditions, must also occur^11,19,64^. For example, in Wilkes Land^65,66,70^, and elsewhere in East Antarctica^53,71^, it is thought that sea-ice can affect the stress conditions of floating ice shelves/tongues by exerting an important buttressing force.

To summarise, our discovery of the widespread development of SGLs around the margins of the EAIS holds important implications for its mass balance and the stability of some of its major outlet glacier catchments. We have shown that surface meltwater is ponding in most peripheral regions of the ice sheet and much further inland and at much higher elevations than previously observed^4^. In this sense, its surface hydrology bears a close resemblance to the GrIS. Our dataset represents an important benchmark that could be used to test whether SGLs will enlarge and perhaps begin to appear at higher elevations, which has recently been observed^21,24^ and modelled^7^ in Greenland, but which is modulated by local albedo effects, ice surface topography and firn properties. Given that the austral summer of 2016/2017 is likely to have been an above-average melt year^35^, future surveys could also seek to better understand the inter-annual variability in SGL development and the extent to which January 2017 may have been unusual. Indeed, our methodological framework facilitates the rapid processing and consistent mapping of thousands of SGLs. Furthermore, the burgeoning availability of imagery and ice velocity datasets should allow regional studies to map lakes at a much higher temporal resolution to search for possible drainage events and ice dynamical effects^25^. Finally, numerical ice sheet models indicate that the storage of meltwater in marginal areas, and particularly ice shelves, is likely to be critical to the future stability of some major marine-based catchments in East Antarctica under a warning climate^54^. Our findings of widespread development of SGLs on floating ice could provide either a test, or constraint, for some of those models, and clearly indicate that some regions of the EAIS may be closer to the threshold of instability than previously thought.

## Methods

### Satellite image acquisition

Our primary source of imagery was from Sentinel 2A, which represents the highest spatial resolution imagery (10 m) that is available free of charge (from the Copernicus Open Access Hub: https://scihub.copernicus.eu/). We acquired all cloud-free scenes available from January 2017, but also included a smaller proportion (~10%) of scenes with minimal cloud cover (<10%) of the marginal areas of the ice sheet. Due to incomplete image coverage and/or scenes with larger amounts of cloud cover, we supplemented the Sentinel 2A imagery with cloud-free scenes from the Landsat-8 Operational Land Imager (OLI), which were acquired free-of-charge from the United States Geological Survey (USGS) Earth Resources Observation Science (EROS) Centre (https://eros.usgs.gov). The majority of lakes were mapped using the Sentinel imagery (~75%), but Landsat was particularly useful to search for lakes further inland and to fill gaps in between Sentinel imagery. The multi-spectral bands of the Landsat-8 images were ‘pan-sharpened’ to 15 m using the panchromatic Band 8 and the High Pass Modulation (HPM) method. This was to bring the spatial resolution of the Landsat imagery closer to that of the Sentinel imagery, but it should be noted that the coarser resolution might be unable to detect some small lakes that would be mapped on the Sentinel imagery (see Errors and Uncertainties, below). In total, we acquired 312 individual scenes totaling almost 5 million km^2^, see Supplementary Fig. 1. The temporal coverage of the imagery is shown in Supplementary Fig. 2. Only ~600 km of the ice sheet’s margin was unable to be observed, largely due to a lack of cloud-free scenes in January 2017, representing ~6% of the perimeter. Images were displayed and manipulated in the Geographic Information Systems (GIS) software ArcMap (http://desktop.arcgis.com), where all subsequent analyses, described below, were undertaken.

### Image processing and automated supraglacial lake identification and mapping

Various approaches have been used to map the area of SGLs on satellite imagery, including manual delineation and a range of automated/semi-automated techniques (e.g. refs^2,4,9,21,23,25,31^). Given the large size of our study area and the large number of scenes to be analysed, we used the semi-automated Normalised Difference Water Index (NDWI). Although most glaciological studies use a variant of the NDWI that includes the blue and red bands^25,33,72,73^, we found that the standard NDWI equation performed very well compared to manual digitization (see Supplementary Figs 3–6). This equation (Eq. 1) was therefore used to classify each image into ‘water’ or ‘no-water’ regions using a threshold value that was interactively selected:

1 $$NDWI=\frac{{\rm{Green}}-{\rm{NIR}}}{{\rm{Green}}+{\rm{NIR}}}$$

where ‘Green’ is Band 3 of the Sentinel imagery and Band 3 of the Landsat 8 imagery, and ‘NIR’ is Band 4 of the Sentinel imagery and Band 5 of the Landsat imagery. Threshold values > 0.2 to >0.5 are commonly used to identify lakes^25,33^, with higher thresholds typically leading to the identification of smaller numbers of lakes (and smaller individual lake areas), and excluding shallower areas or areas of slush; and with lower thresholds tending to identify larger numbers of lakes (and larger lake areas), and potentially including shallow lakes and areas of slush. In this study, we experimented with a number of thresholds within this range and found a value of >0.3 performed most accurately compared to manual digitization (see Supplementary Figs 4–6). This value is similar to the threshold of 0.25 to identify ponds on the Nansen Ice Shelf, East Antarctica^33^. In addition to quantitative comparisons with manual delineation techniques, we also undertook visual comparisons between lake outlines and the underlying imagery. We found that our threshold prevented any obvious misclassifications with blue-ice areas, which cover around 1.7% of Antarctica^74^ (see Errors and Uncertainties below).

The NDWI assigns each pixel a value, which was classified as ‘lake’ or ‘no lake’ using our threshold of >0.3. Following previous work^24,31^, and to reduce noise in the initial output, we used a minimum size-threshold of two pixels for lake detection, which gives a minimum lake size of 200 m^2^ for Sentinel and 450 m^2^ for Landsat imagery. Pixels classified as lakes in a raster image were then converted to a vector format (shapefile) in ArcGIS (Supplementary Figs 3 and 4). To further reduce noise and improve the visual clarity of the dataset, we then applied a majority filter to generalize the edge of polygons, and used the dissolve function to clean up overlapping pixels. We then applied the aggregate tool to combine pixels within a distance of 20 m (2 pixel resolution in the Sentinel data). Apart from potentially reducing large numbers of very, very small SGLs (<200 m^2^), these changes were largely cosmetic and are insignificant in terms of the uncertainties associated with identifying lakes and calculating total lake area, described in the next section. The lake outlines are available as GIS shapefiles (.shp) in Supplementary Data 1 and all data used in our analysis are available in a spreadsheet in Supplementary Data 2.

### Quantification of errors and uncertainties in supraglacial lake mapping

Initially, we performed a visual cross-check of the lake polygons against the underlying imagery and removed any obvious false positives (less than a few hundred), which were mainly associated with nunataks, blue ice areas^74^ or areas of shadow cast by nunataks. We then quantified the uncertainties of our automated mapping technique by comparing our results with those obtained from manual digitization. Three sample areas containing a variety of SGL shapes and sizes deemed to be broadly representative of the range of lake characteristics across the ice sheet were selected. The boundaries of lakes were manually digitized on screen making full use of various band combinations and varying the scale to suit the size of the lake (see Supplementary Fig. 4). We then compared the manually-digitized lake areas to those calculated from the automated NDWI method (e.g. Supplementary Fig. 5) and found very close agreement between the two methods (Supplementary Fig. 6). In general, manual digitizing tended to generate slightly larger individual lake areas, but this varied according to the size of individual lakes (Supplementary Fig. 6), with the smallest lakes (<0.01 km^2^) generating the largest percentage differences, but where the absolute area differences were obviously much smaller. For larger lakes, the two methods were much more similar. However, the differences between the total lake area for each of the three sample areas (Supplementary Fig. 4) were all <0.5%. We therefore conclude that whilst individual lake areas might vary using each method, especially for very small (<0.01 km^2^) lakes, the total area of lakes mapped across much larger areas is likely to be very similar for each technique and we assign a conservative uncertainty of 1% to our total lake area of 1,383 km^2^. It is also worth noting that varying the threshold that was applied to the NDWI output (but without obviously misclassifying lakes) was within the range gleaned from the manual versus automated digitizing uncertainty.

Quantifying the uncertainty regarding lake identification (as opposed to lake area) and, therefore, the total number of lakes is much more difficult, especially with limited ‘ground truth’ data. Having compared our mapped lake outlines with imagery and removed obvious ‘false positives’, we are confident that there are very few (if any) non-lake areas in our dataset. However, it is much more difficult to ascertain and quantify how many lakes might be missing from our January 2017 dataset, especially very small lakes.

Firstly, it is important to note that it was not possible to acquire all imagery on the same day and so we had to sample/observe imagery from different days in January (see Supplementary Fig. 2). In areas where we had several images available, we sampled the image closest to the middle of January around the likely peak of the melt season. This means that we may have missed some lakes that developed relatively late in the melt season in some areas, but this may be counteracted by early ‘freeze-up’ in other areas. Moreover, in some regions we only had images available from early January (when lakes may yet have reached their full extent) or late January (when lakes may have started to freeze over). Thus, our dataset is likely to be representative of the broad distribution and surface area of lakes across the ice sheet in mid-January, but that the number of lakes and their total area, should probably be viewed as a minimum for the peak of the melt season in January 2017.

Secondly, it is likely that some very small and/or shallow lakes were not identified (e.g. <0.0002 km^2^), especially where we were restricted to using the 15 m Landsat 8 imagery. We sampled several small areas where some of the smallest lakes in our dataset were clustered. In these regions, there is often a transition from what is likely to be saturated firn to small, shallow ponds, and to larger and more obvious lakes (see also ref.^33^). Thus, it is very difficult to work to a binary definition of ‘lake’ versus ‘non-lake’ in these areas, especially given the resolution of the imagery (field observations would encounter similar issues). It is for this reason that we use a lower size threshold of 0.0002 km^2^ (200 m^2^) and we performed some of the statistical filters to clean and generalize our SGL polygons. This is another reason why we view our estimate of the total number of lakes as a minimum and why their cumulative surface area is also likely to be a minimum. That said, even if we had missed ~25% of the total number of lakes identified in our dataset (i.e. ~16,000) that existed at our minimum size limit (0.0002 km^2^), they would together only add 3.2 km^2^ to our total area of 1,383 km^2^ (just 0.2%). We also compared the results from the automated mapping of the same area to test the impact of image resolution. Clearly, mapping from Sentinel 2A imagery enabled the detection of smaller lakes (minimum of 200 m^2^) compared to the Landsat 8 (450 m^2^), but the agreement was generally excellent for lakes >450 m^2^. Overall, we suggest that whilst our estimate of the number of lakes is a only a minimum, any missing lakes (e.g. not mapped in the Landsat imagery) are unlikely to add substantially to the total area of 1,383 km^2^ and this certainly falls well below our total lake area uncertainty of 1% (13.8 km^2^). Moreover, the absolute number of lakes is less useful/important than their distribution and cumulative area (e.g. when comparing patterns across the ice sheet and between different regions).

### Estimating supraglacial lake volumes

Although algorithms exist to calculate the depth/volume of SGLs and have been used successfully in smaller study areas (and with generally small numbers of satellite scenes)^25,58–60^, there are additional complications when attempting to consistently extract lake depth from hundreds of scenes across two different sensors. Moreover, our study is primarily focused on the distribution and surface area of lakes across the whole ice sheet. Nevertheless, in order to provide a simple first order approximation of the likely volume of each SGL, we regressed lake area against lake volume for two Antarctic datasets from the pre-collapse Larsen B ice shelf on the Antarctic Peninsula^56^ (n = 8,398) and Langhovde Glacier in East Antarctica^31^ (n = 1,738). We acknowledge that these two settings may not be representative of the full spectrum of lakes in our dataset, but they reveal a strong and significant relationship (r^2^ = 0.86) between area (*A*) and volume (*V*), albeit with greater scatter of volumes at lower areas (Supplementary Fig. 7). We use this regression to derive an area-volume scaling (Eq. 2), which was then applied to our dataset to estimate the likely range of volumes (Supplementary Fig. 8) where:

2 $$V=7.16{e}^{-4}A$$

Similarly strong area-volume scaling relationships have been reported from several studies in Greenland that suggest that lake volume is highly dependent on lake area^22,25,57^.

### Extracting supraglacial lake characteristics from ice sheet topography and velocity data

Each SGL was represented by a polygon within a single vector shapefile (e.g. Supplementary Fig. 4) and we used the GIS software (ArcMap) to automatically extract the location (geometric centroid of each lake polygon, using a Polar Stereographic projection) and surface area of each lake. To extract data on the ice sheet surface topography associated with each lake, we used the Bedmap2 continent-wide dataset of ice thickness (gridded at 1 km resolution)^38^. This allowed us to extract values of surface elevation at the geometric centre of each SGL. We also converted the ice surface topography into a slope map to extract the surface slope of the ice sheet topography at the location of each lake. Bedmap2 also provides a ‘rock mask’, which we used to calculate the planar distance of the nearest edge of each lake polygon to the nearest edge of exposed rock. Closer inspection of the rock mask dataset indicated that some very small nunataks were missing (due to the spatial resolution of the Bedmap2 dataset) and so a small number of the lakes might be nearer to bedrock than we measured, i.e. distances presented in Figs 3f and 6 are likely to be maximum distances to bedrock. Similar analyses were undertaken to calculate the distance of each lake to the coastline and the distance of each lake to the grounding line, both of which were acquired from the Bedmap2 study. We are aware that the Bedmap2 grounding line has been updated and the accuracy improved in some regions, but it remains the only consistent pan-ice-sheet product and recent updates/changes to grounding line positions are insignificant given the broad aims of our continent-wide survey (e.g. our 5 km bin range in Fig. 3d). Finally, we also extracted the ice surface velocity at the location (geometric centroid) of each lake from a recently published dataset^75^ (gridded at 450 m) based on satellite radar interferometry (2007–2009).

Note that 1,716 lakes were located beyond the ice margin provided in the Bedmap2 dataset. This appeared to be due to changes in the ice margin (i.e. advance of ice tongues/ice shelves) since the compilation of the Bedmap2 dataset. Thus, we did not analyse the physiographic characteristics of this small percentage (2.6%) of the overall population of lakes, although we do include them in our calculations of the number and area of lakes.

### Comparison to ice shelf vulnerability to hydrofracture

A recent study^11^ quantified an ice shelf vulnerability index for Antarctica based on the relationship between active microwave backscatter data and average annual melt days. This was based on field data from a transect in Greenland that demonstrated that backscatter values increased with increasing mean annual melt days until specular reflections due to large, continuous ice lenses in the firn layer at very high melt days causes the backscatter to decrease. They used these relationships to create an index that quantified ice shelf vulnerability to surface-melt-induced collapse via hydrofracture, which was then applied to Antarctica. This assumed that the ice shelf backscatter/melt days relationship represents the temporal evolution that any given ice shelf might experience under changing climate conditions. This allowed them to identify which ice shelves are currently, or may soon be, vulnerable to hydrofracture. We compared our data on lake area density (total lake area per 15 km^2^) to this previously-published vulnerability index (ranging from 0 to 1, with 1 indicating high vulnerability). The vulnerability data were supplied in tagged image format (.tif) and gridded at 4.45 km resolution, which we ingested into our GIS for directly comparison (see Fig. 7).

## Supplementary information

Supplementary Information

Supplementary Dataset 1

Supplementary Dataset 2

## Data Availability

The mapped supraglacial lake polygons are available as digital GIS shapefiles (.shp) in Supplementary Data 1. A spreadsheet of supraglacial lake characteristics (in Microsoft Excel format) is available in Supplementary Data 2.

## References

[1] Echelmeyer K, Clarke TS, Harrison WD (1991). Surficial glaciology of Jaskobshavns Isbrae, West Greenland. J. Glaciol..

[2] Box JE, Ski K (2007). Remote sounding of Greenland supraglacial melt lakes: implications for subglacial hydraulics. J. Glaciol..

[3] Banwell AF (2014). Supraglacial lakes on the Larsen B ice shelf, Antarctica, and at Paakitsoq, West Greenland: a comparative study. Ann. Glaciol..

[4] Kingslake J, Ely JC, Das I, Bell RE (2017). Widespread movement of meltwater onto and across Antarctic ice shelves. Nature.

[5] Lüthje M, Pedersen LT, Reeh N, Greull W (2006). Modelling the evolution of supraglacial lakes on the West Greenland ice-sheet margin. J. Glaciol..

[6] Tedesco M (2012). Measurement and modeling of ablation of the bottom of supraglacial lakes in western Greenland. Geophys. Res. Lett..

[7] Leeson AA (2015). Supraglacial lakes on the Greenland ice sheet advance inland under warming climate. Nat. Clim. Change..

[8] Scambos TA, Hulbe C, Fahnestock M, Bohlander J (2000). The link between climate warming and break-up of ice shelves in the Antarctic Peninsula. J. Glaciol..

[9] Glasser NF, Scambos TA (2008). A structural glaciological analysis of the 2002 Larsen B ice-shelf collapse. J. Glaciol..

[10] Banwell AF, MacAyeal DR, Sergienko OV (2013). Breakup of the Larsen B Ice Shelf triggered by chain reaction drainage of supraglacial lakes. Geophys. Res. Lett..

[11] Alley KE, Scambos TA, Miller JZ, Long DG, MacFerrin M (2018). Quantifying vulnerability of Antarctic ice shelves to hydrofracture using microwave scattering properties. Rem. Sens. Env..

[12] Banwell AF, Willis IC, Macdonald GJ, Goodsell B, MacAyeal DR (2019). Direct measurements of ice-shelf flexure caused by surface meltwater ponding and drainage. Nat. Commun..

[13] De Angelis H, Skvarca P (2003). Glacier surge after ice shelf collapse. Science.

[14] Rignot E (2004). Accelerated ice discharge from the Antarctic Peninsula following the collapse of the Larsen B Ice Shelf. Geophys. Res. Lett..

[15] Zwally HJ (2002). Surface melt-induced acceleration of Greenland ice-sheet flow. Science.

[16] Das SB (2008). Fracture propagation to the base of the Greenland Ice Sheet during supraglacial lake drainage. Science.

[17] Bartholomew I (2010). Seasonal evolution of subglacial drainage and acceleration in a Greenland outlet glacier. Nat. Geosci..

[18] Doyle SH (2013). Ice tectonic deformation during the rapid *in situ* drainage of a supraglacial lake on the Greenland Ice Sheet. Cryosphere.

[19] Stevens LA (2015). Greenland supraglacial lake drainages triggered by hydrologically induced basal slip. Nature.

[20] Bell RE, Banwell AF, Trusel LD, Kingslake J (2018). Antarctic surface hydrology and impacts on ice-sheet mass balance. Nat. Clim Change.

[21] Sundal AV (2009). Evolution of supra-glacial lakes across the Greenland Ice Sheet. Rem. Sens. Env..

[22] Liang Y-L (2012). A decadal investigation of supraglacial lakes in West Greenland using a fully automatic detection and tracking algorithm. Rem. Sens. Env..

[23] Leeson AA (2013). A comparison of supraglacial lake observations derived from MODIS imagery at the western margin of the Greenland ice sheet. J. Glaciol..

[24] Howat IM, de la Peña S, van Angelen JH, Lenaerts JTM, van den Broeke MR (2013). Brief Communication: “Expansion of meltwater lakes on the Greenland Ice Sheet”. Cryosphere.

[25] Williamson AG, Arnold NS, Banwell AF, Willis IC (2017). A Fully Automated Supraglacial lake area and volume Tracking (“FAST”) algorithm: Development and application using MODIS imagery of West Greenland. Rem. Sens. Env..

[26] Zwally, H. J. & Fiegles, S. Extent and duration of Antarctic surface melt. *J. Glaciol*. **40**, 463–477 (1994).

[27] Trusel LD, Frey KE, Das SB (2012). Antarctic surface melting dynamics: enhanced perspectives from radar scatterometer data. J. Geophys. Res..

[28] Trusel LD (2013). Satellite-based estimates of Antarctic surface meltwater fluxes. Geophys. Res. Lett..

[29] Lenaerts JTM, Vizcaino M, Fyke J, van Kampenhout L, van den Broeke MR (2016). Present-day and future Antarctic ice sheet climate and surface mass balance in the Community Earth System Model. Clim. Dyn..

[30] Kingslake J, Ng F, Sole A (2015). Modelling channelized surface drainage of supraglacial lakes. J. Glaciol..

[31] Langley Emily S., Leeson Amber A., Stokes Chris R., Jamieson Stewart S. R. (2016). Seasonal evolution of supraglacial lakes on an East Antarctic outlet glacier. Geophysical Research Letters.

[32] Lenaerts JTM (2017). Meltwater produced by wind-albedo interaction stored in an East Antarctica ice shelf. Nat. Clim. Change..

[33] Bell RE (2017). Antarctic ice shelf potentially stabilized by export of meltwater in surface river. Nature.

[34] Trusel LD (2015). Divergent trajectories of Antarctic surface melt under two twenty-first-century climate scenarios. Nat. Geosci..

[35] Turner J (2017). Unprecedented springtime retreat of Antarctic sea ice in 2016. Geophys. Res. Lett..

[36] Leppäranta M, Järvinen O, Mattila O-P (2013). Structure and life cycle of supraglacial lakes in Dronning Maud Land. Antarct Sci..

[37] Phillips HA (1998). Surface melt-streams on the Amery Ice Shelf, east Antarctica. Ann. Glaciol..

[38] Fretwell P (2013). Bedmap2: improved ice bed, surface and thickness datasets for Antarctica. Cryosphere.

[39] Winther J-G, Elvehøy H, Bøggild CE, Sand K, Liston G (1996). Melting, runoff and the formation of frozen lakes in a mixed snow and blue-ice field in Dronning Maud Land, Antarctica. J. Glaciol..

[40] Kuipers Munneke P, Picard G, van den Broeke MR, Lenaerts JTM, Meijgaard E (2012). Insignificant change in Antarctic snowmelt volume since 1979. Geophys. Res. Lett..

[41] Kuipers Munneke P, Ligtenberg SRM, van den Broeke MR, Vaughan DG (2014). Firn air depletion as a precursor of Antarctic ice-shelf collapse. J. Glaciol..

[42] Van Wessem JM (2018). Modelling the climate and surface mass balance of polar ice sheets using RACMO2 – Part 2: Antarctica (1979-2016). Cryosphere.

[43] LaBarbera CH, MacAyeal DR (2011). Traveling supraglacial lakes on George VI Ice Shelf, Antarctica. Geophys. Res. Lett..

[44] Dow CF (2018). Basal channels drive active surface hydrology and transverse ice shelf fracture. Sci. Adv..

[45] McGrath D (2012). Basal crevasses on the Larsen C Ice Shelf, Antarctica: Implications for meltwater ponding and hydrofracture. Geophys. Res. Lett..

[46] Gudmundsson GH (2003). Transmission of basal variability to a glacier surface. J. Geophys. Res..

[47] Glasser NF, Gudmundsson GH (2012). Longitudinal surface structures (flowstripes) on Antarctic glaciers. Cryosphere.

[48] Ely J. C., Clark C. D., Ng F. S. L., Spagnolo M. (2017). Insights on the formation of longitudinal surface structures on ice sheets from analysis of their spacing, spatial distribution, and relationship to ice thickness and flow. Journal of Geophysical Research: Earth Surface.

[49] Leeson AA (2017). Regional climate of the Larsen B embayment 1980–2014. J. Glaciol..

[50] Lenaerts JTM, van den Broeke MR, van de Berg WJ, van Meijgaard E, Kuipers Munneke P (2012). A new, high-resolution surface mass balance map of Antarctica (1979–2010) based on regional atmospheric climate modeling. Geophys. Res. Lett..

[51] Polashenski C (2014). Observations of pronounced Greenland ice sheet firn warming and implications for runoff prediction. Geophys. Res. Lett..

[52] Hubbard B (2016). Massive subsurface ice formed by refreezing of ice-shelf melt ponds. Nat. Commun..

[53] Liang Q (2019). Ice flow variations at Polar Record Glacier, East Antarctica. J. Glaciol..

[54] DeConto RM, Pollard D (2016). Contribution of Antarctica to past and future sea-level rise. Nature.

[55] Ligtenberg SRM, Kuipers Munneke P, van den Broeke MR (2014). Present and future variations in Antarctic firn air content. Cryosphere.

[56] Leeson AA, Foster E, Rice A, Gourmelen N, van Wessem M (2019). Quantifying the evolution of supraglacial lakes on Larsen B Ice Shelf in the two decades preceding its collapse, using spaceborne optical and SAR data. Geophysical Research Abstracts.

[57] Morris BF (2013). A ten-year record of supraglacial lake evolution and rapid drainage in West Greenland using an automated processing algorithm for multispectral imagery. Cryosphere.

[58] Sneed WA, Hamilton GS (2007). Evolution of melt pond volume on the surface of the Greenland Ice Sheet. Geophys. Res. Lett..

[59] Pope A (2016). Reproducibility estimating and evaluating supraglacial lake depth with Landsat 8 and other multispectral sensors. Earth and Space Science.

[60] Pope A (2016). Estimating supraglacial lake depth in West Greenland using Landsat 8 and comparison with other multispectral methods. Cryosphere.

[61] Van der Veen CJ (1998). Fracture mechanics approach to penetration of surface crevasses on glaciers. Cold Reg. Sci. Technol..

[62] MacAyeal DR, Sergienko OV (2013). Flexural dynamics of melting ice shelves. Ann. Glaciol..

[63] Dupont TK, Alley RB (2005). Assessment of the importance of ice-shelf buttressing to ice-sheet flow. Geophys. Res. Lett..

[64] Fürst JJ (2016). The safety band of Antarctic ice shelves. Nat. Clim. Change..

[65] Miles BWJ, Stokes CR, Jamieson SSR (2016). Pan-ice sheet glacier terminus change in East Antarctica reveals sensitivity of Wilkes Land to sea-ice changes. Sci. Adv..

[66] Miles BWJ, Stokes CR, Jamieson SSR (2017). Simultaneous distintegration of outlet glaciers in Porpoise Bay (Wilkes Land), East Antarctica, driven by sea ice break-up. Cryosphere.

[67] Rignot E (2019). Four decades of Antarctic Ice Sheet mass balance from 1979–2017. Proc. Natl. Acad. Sci..

[68] Pittard ML, Galton-Fenzi BK, Watson CS, Roberts JL (2017). Future sea level change from Antarctica’s Lambert-Amery glacial system. Geophys. Res. Lett..

[69] Christoffersen P (2018). Cascading lake drainage on the Greenland Ice Sheet triggered by tensile shock and fracture. Nat. Commun..

[70] Greene CA (2018). Seasonal dynamics of Totten Ice Shelf controlled by sea ice buttressing. Cryosphere.

[71] Miles BWJ, Stokes CR, Jamieson SSR (2018). Velocity increases at Cook Glacier, East Antarctica, linked to ice shelf loss and a subglacial flood event. Cryosphere.

[72] Yang K, Smith LC (2013). Supraglacial streams on the Greenland Ice Sheet delineated from combined spectral-shape information in high-resolution satellite imagery. IEEE Geosci Remote S..

[73] Moussavi MS (2016). Derivation of supraglacial lake volumes on the Greenland Ice Sheet from high resolution satellite imagery. Rem. Sens. Env..

[74] Hui F (2014). Mapping blue-ice areas in Antarctica using ETM+ and MODIS data. Ann. Glaciol..

[75] Rignot E., Mouginot, J. & Scheuchl, B. MEaSUREs InSAR-Based Antarctica Ice Velocity Map, http://nsidc.org/data/NSIDC-0484 (National Snow and Ice Data Center, 2011).

