# Author Correction: Widespread distribution of supraglacial lakes around the margin of the East Antarctic Ice Sheet

**DOI:** 10.1038/s41598-020-58950-3

**Published:** 2020-01-31

**Authors:** Chris R. Stokes, Jack E. Sanderson, Bertie W. J. Miles, Stewart S. R. Jamieson, Amber A. Leeson

**Affiliations:** 1 Department of Geography, Durham University, Durham, DH1 3LE UK; 2 Lancaster Environment Centre/Data Science Institute, Lancaster University, Bailrigg, Lancaster, LA1 4YW UK

Correction to: *Scientific Reports* 10.1038/s41598-019-50343-5, published online 25 September 2019

This Article contains a typographical error in the Methods section under subheading ‘Image processing and automated supraglacial lake identification and mapping’ where,

“This equation (Eq. 1) was therefore used to classify each image into ‘water’ or ‘no-water’ regions using a threshold value that was interactively selected:

$$NDWI=\frac{Green-NIR}{Green+NIR}$$

where ‘Green’ is Band 3 of the Sentinel imagery and Band 3 of the Landsat 8 imagery, and ‘NIR’ is Band 4 of the Sentinel imagery and Band 5 of the Landsat imagery”

should read:

“This equation (Eq. 1) was therefore used to classify each image into ‘water’ or ‘no-water’ regions using a threshold value that was interactively selected:

$$NDWI=\frac{Green-NIR}{Green+NIR}$$

where ‘Green’ is Band 3 of the Sentinel imagery and Band 3 of the Landsat 8 imagery, and ‘NIR’ is Band 8 of the Sentinel imagery and Band 5 of the Landsat imagery”

