# Supplementary material for: Widespread distribution of supraglacial lakes around the margin of the East Antarctic Ice Sheet
Source: Sci Rep. 2019 Sep 25;9:13823. doi: 10.1038/s41598-019-50343-5 (PMC6761196; doi:10.1038/s41598-019-50343-5)
Supplement: Supplementary file 1 — Supplementary Information [file 41598_2019_50343_MOESM1_ESM.pdf]

# Widespread distribution of supraglacial lakes around the margin of the East Antarctic Ice Sheet

Chris R Stokes[1*], Jack E. Sanderson[1], Bertie W.J. Miles[1], Stewart S.R. Jamieson[1], Amber A. Leeson[2]

[1]Department of Geography, Durham University, Durham, DH1 3LE, UK

[2]Lancaster Environment Centre/Data Science Institute, Lancaster University, Bailrigg, Lancaster, LA1 4YW, UK

*Correspondence to: c.r.stokes@durham.ac.uk

**Supplementary Figures 1 to 8**

**Supplementary Data 1**: Digital GIS shapefile (.shp) of supraglacial lake dataset *[to be made available as supplementary data upon publication]*

**Supplementary Data 2**: Digital spreadsheet of supraglacial lake dataset (in Microsoft Excel format) *[to be made available as supplementary data upon publication]*

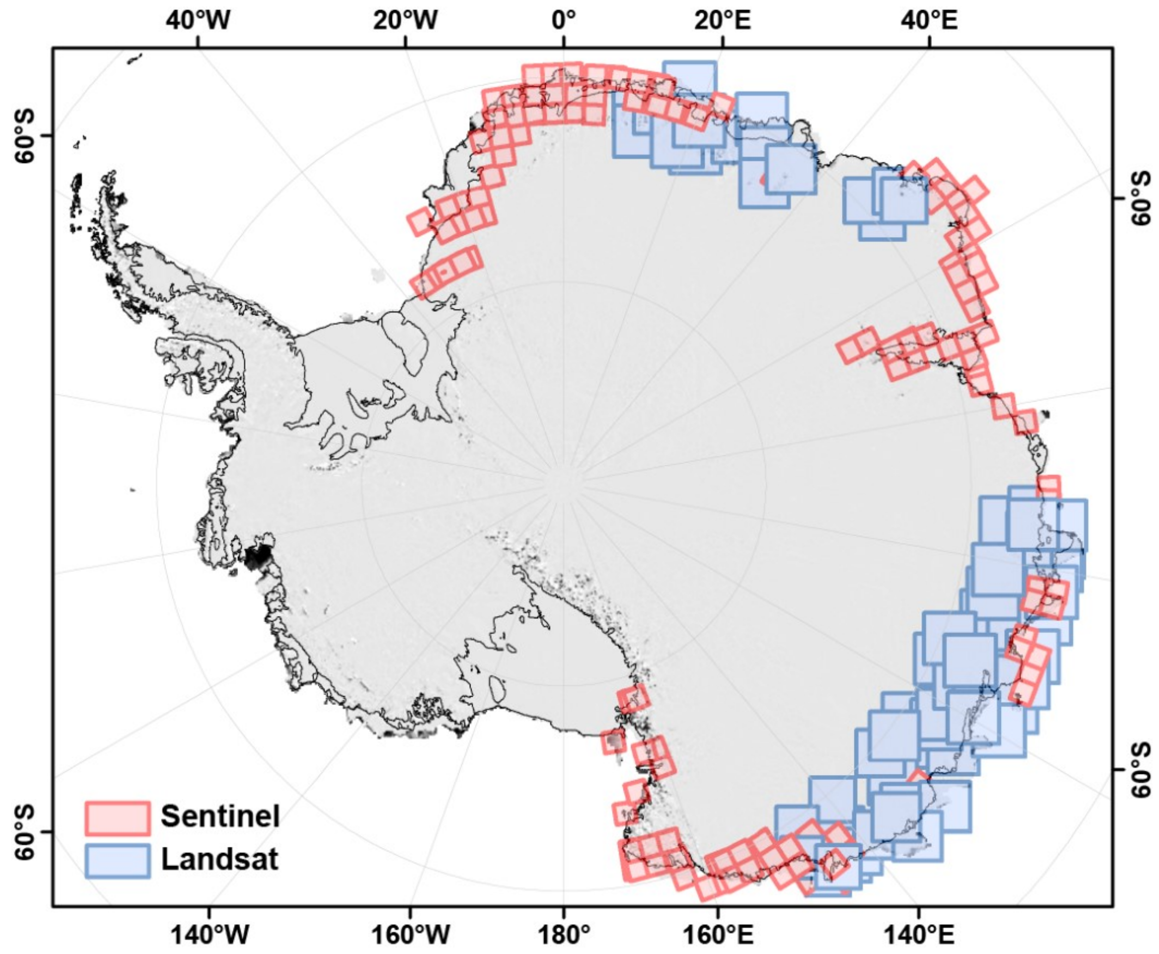


**Supplementary Figure 1: The spatial coverage of the Sentinel 2A and Landsat 8 satellite imagery from January 2017 used in this study** (see Supplementary Fig. 2 for temporal coverage). The lack of data in some areas (6% of the ice sheet perimeter) is largely due to a lack of cloud-free images.

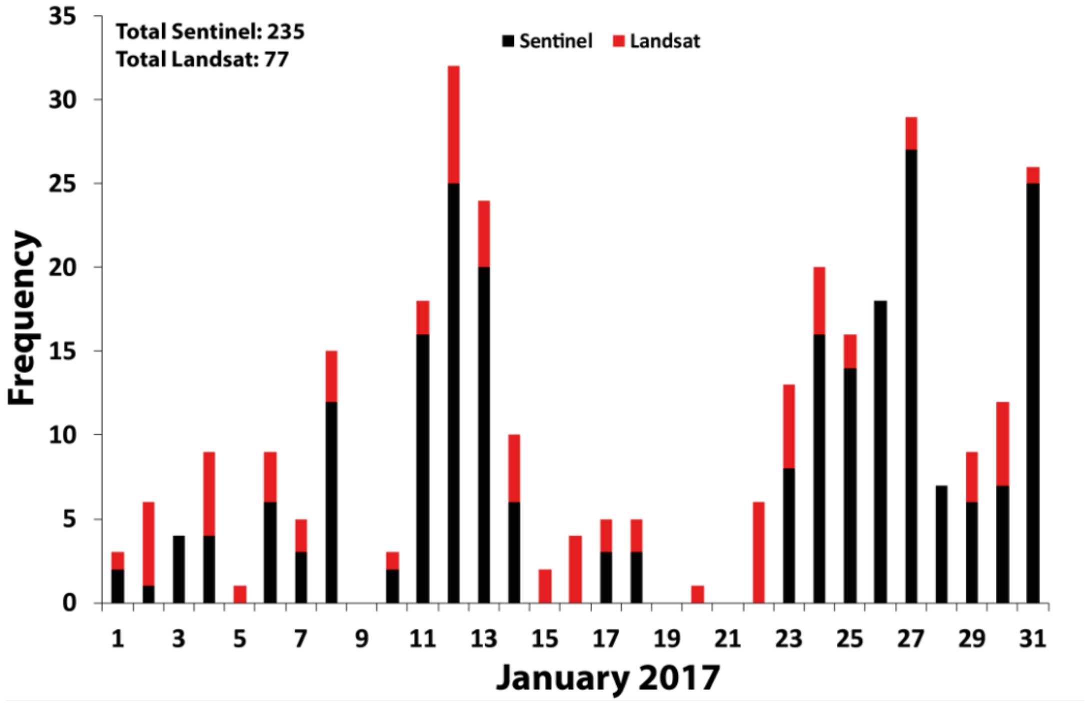


**Supplementary Figure 2: The temporal coverage of the 312 satellite images in this study** (see Supplementary Fig. 1 for spatial coverage).

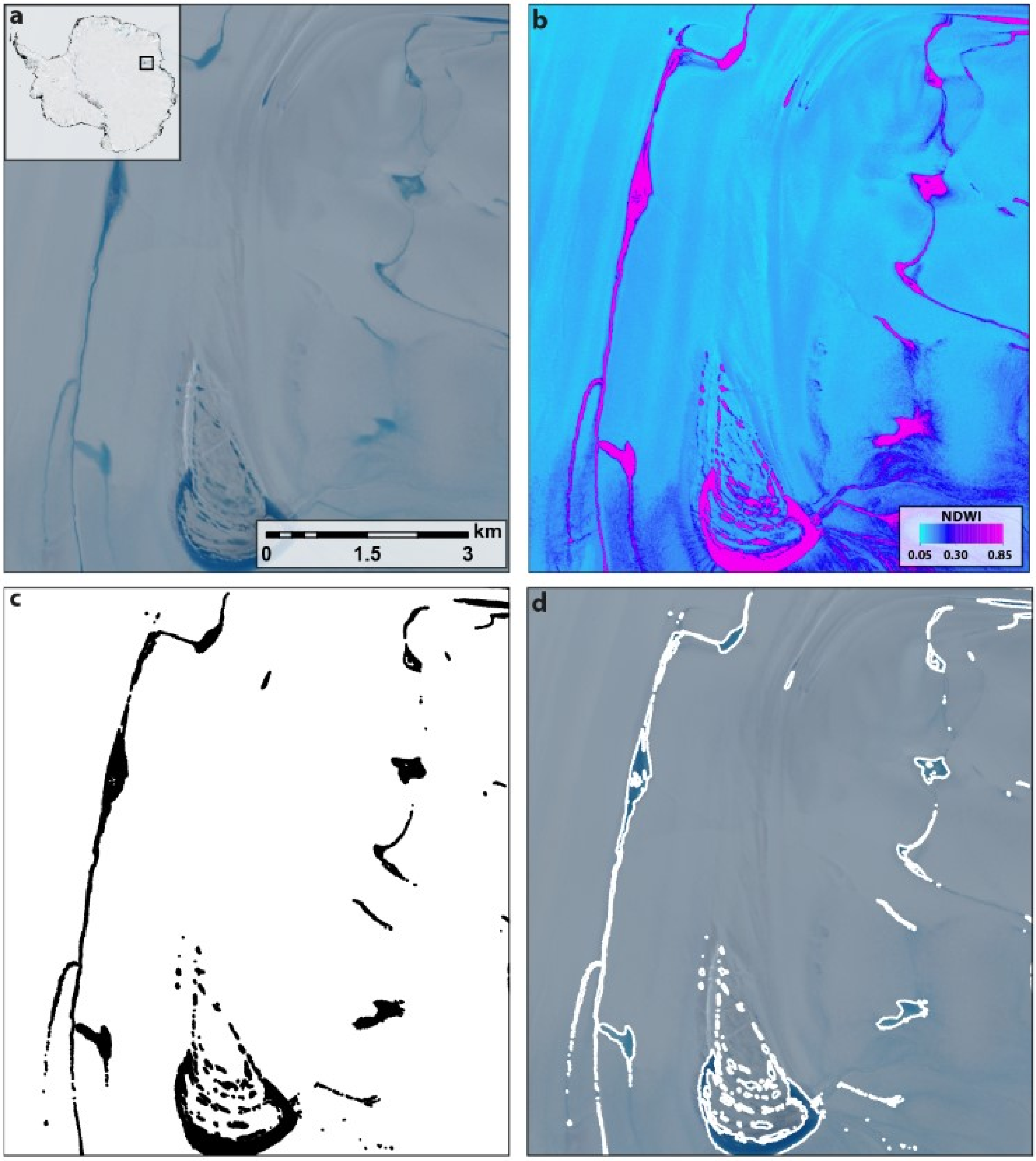


**Supplementary Figure 3: Illustration of the NDWI classification in a complex area of supraglacial lakes and surface meltwater channels.** (**a**) Sentinel 2A satellite image (8$^{th}$ January 2017) of lakes and channels on the Amery Ice Shelf (approximate location shown on inset map). (**b**) Output from the NDWI method, showing surface ponding of meltwater (lakes and channels) identifiable at higher values, e.g. >0.3. (**c**) Binary classification of surface meltwater ponding using a threshold of >0.3 on the image in (b), with surface meltwater shown in black. (**d**) Classification of surface meltwater (white outlines) overlain on the original image from (**a**).

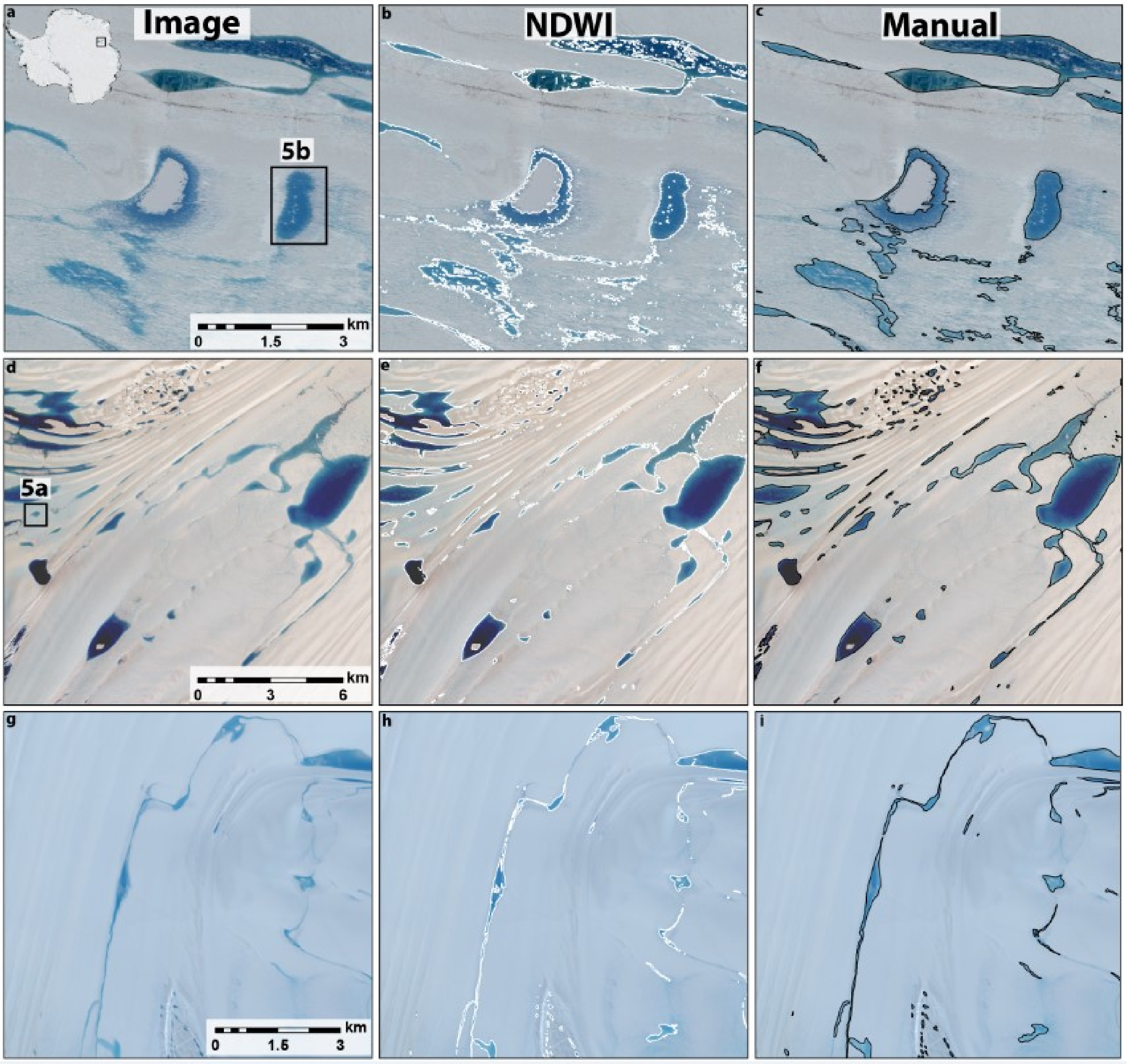


**Supplementary Figure 4: Illustration of the NDWI classification versus manual digitization from three test areas.** (**a, d, g**) show Sentinel 2A satellite images of lakes on the Amery Ice Shelf (location shown in the inset) acquired on 27$^{th}$ January 2017 (**a**), 28$^{th}$ January 2017 (**d**), and 8$^{th}$ January 2017 (**g**). (**b, e, h**) show classification of surface meltwater ponding using the NDWI classification. (**c, f, i**) show the classification of surface meltwater ponding using manual digitizing. Note that automated techniques will tend to generate much larger numbers of smaller lakes, compared to the manual method, but total areas are comparable. The location of the zoomed-in areas on Supplementary Figure 5 are marked. See also Supplementary Figure 6 for a quantitative comparison between the results of the NDWI method and the results of manual digitization.

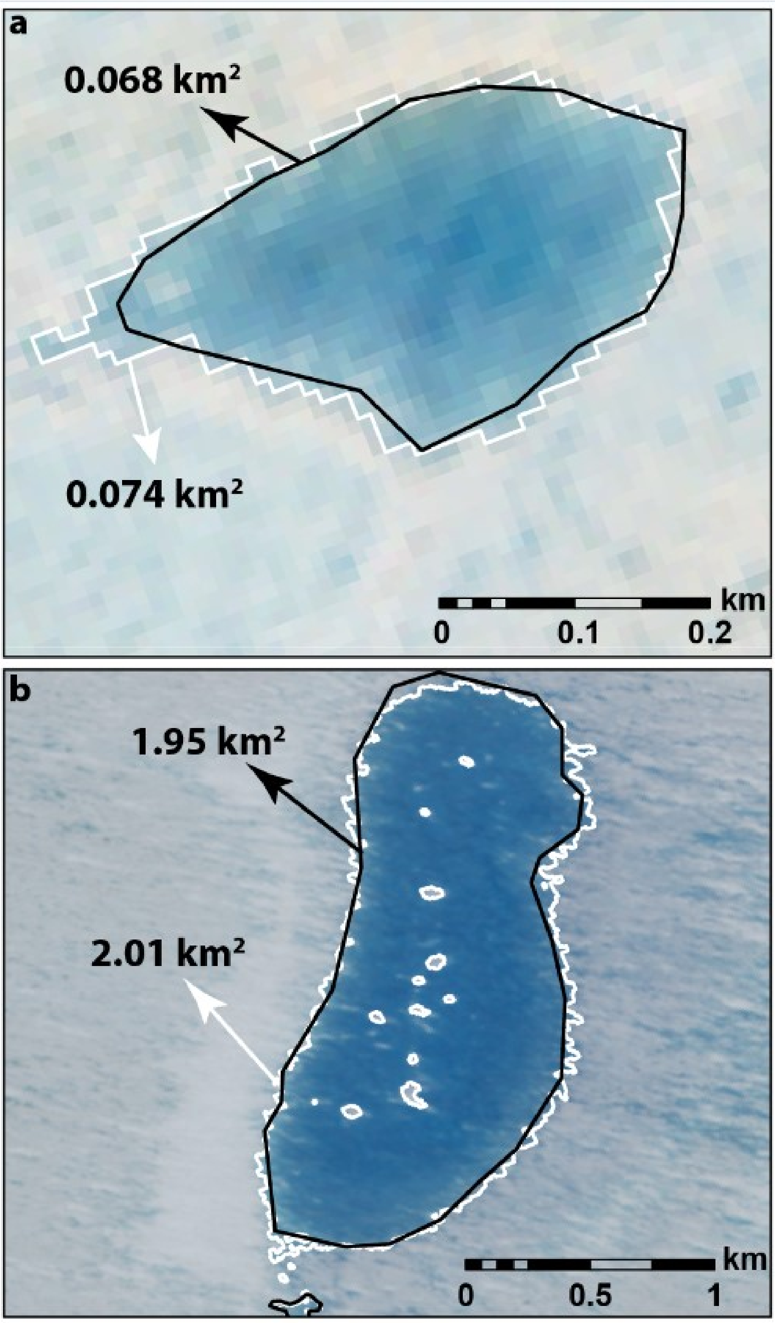


**Supplementary Figure 5: Illustration of the NDWI classification (white outlines) versus manual digitization (black outlines) for a small lake in (a) and larger lake in (b).** Locations are shown on Supplementary Figure 4. See also Supplementary Figure 6 for a quantitative comparison between the results of the NDWI method and the results of manual digitization.

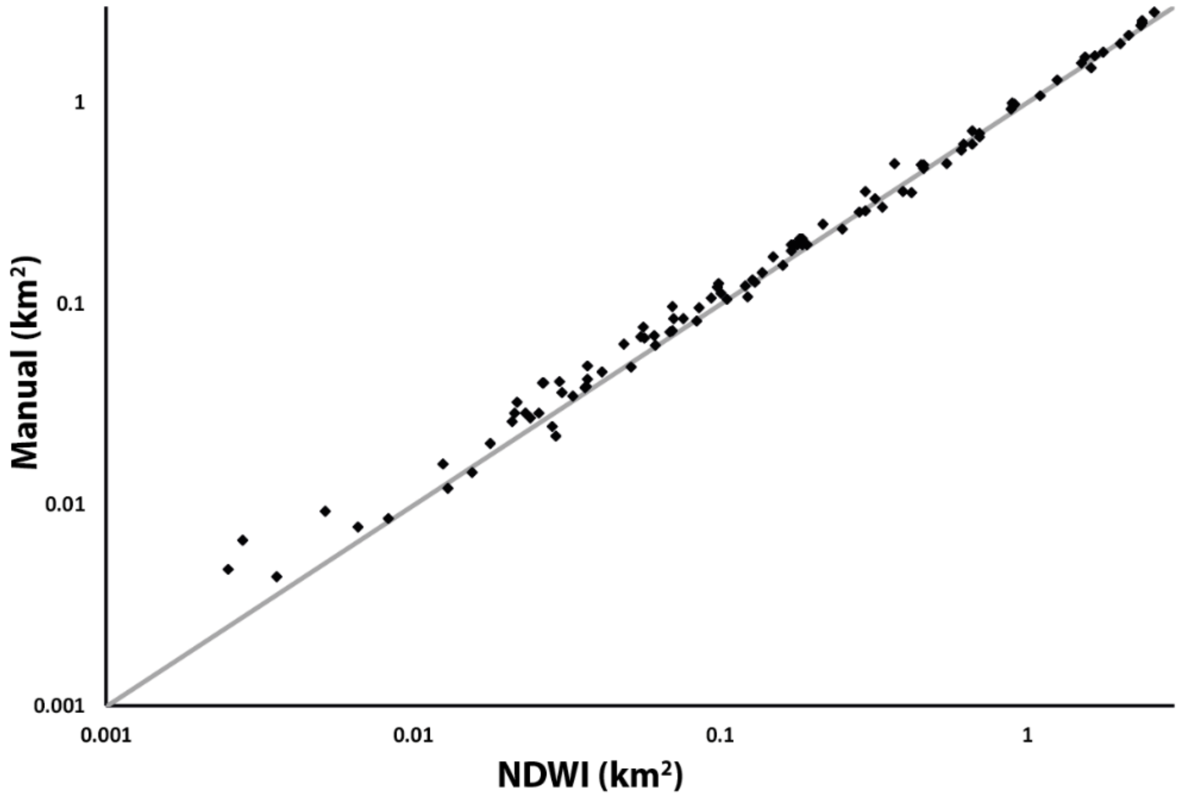


**Supplementary Figure 6: Scatterplot of lake outlines comparing those derived from the automated Normalised Difference Water Index (NDWI) to those derived from manual delineation** (see also Supplementary Fig. 4 and Supplementary Fig. 5). Note the strong correlation of the linear regression ($R^2$ = 0.997), but with a higher scatter at very low lake areas.

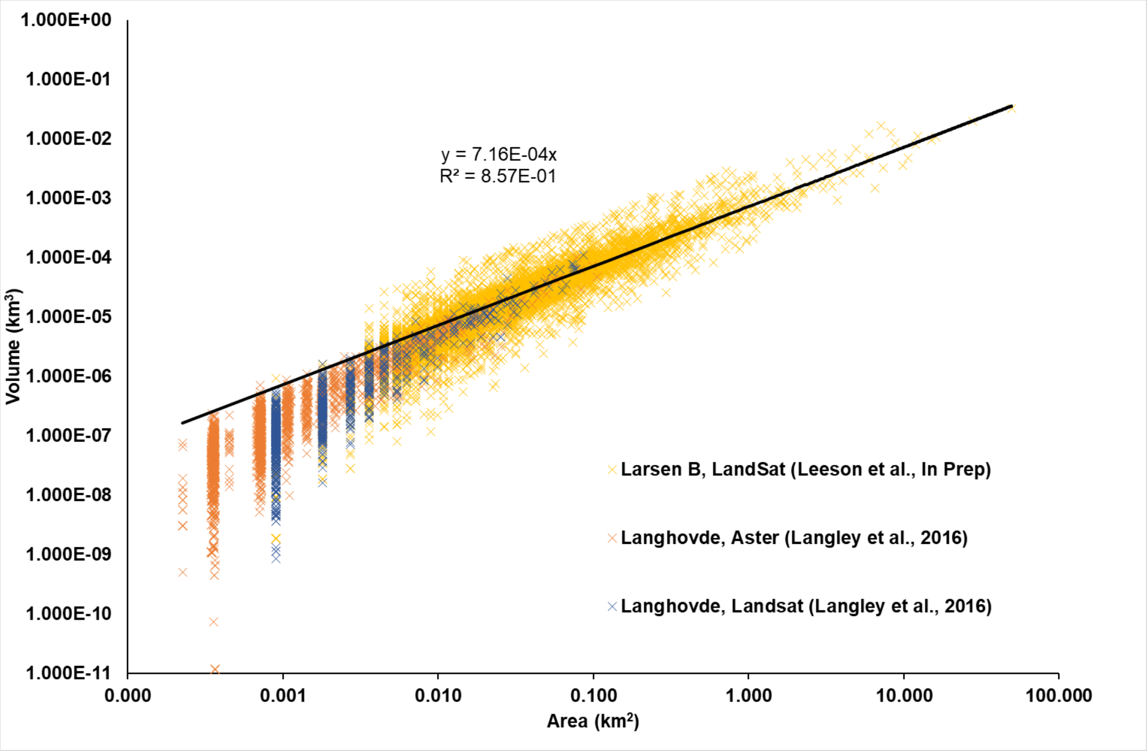


**Supplementary Figure 7: Linear regression of SGL area and volume from two previously measured datasets in Antarctica.** We use this simple scaling relationship to estimate SGL volumes from our measurements of area (Supplementary Fig. 8).

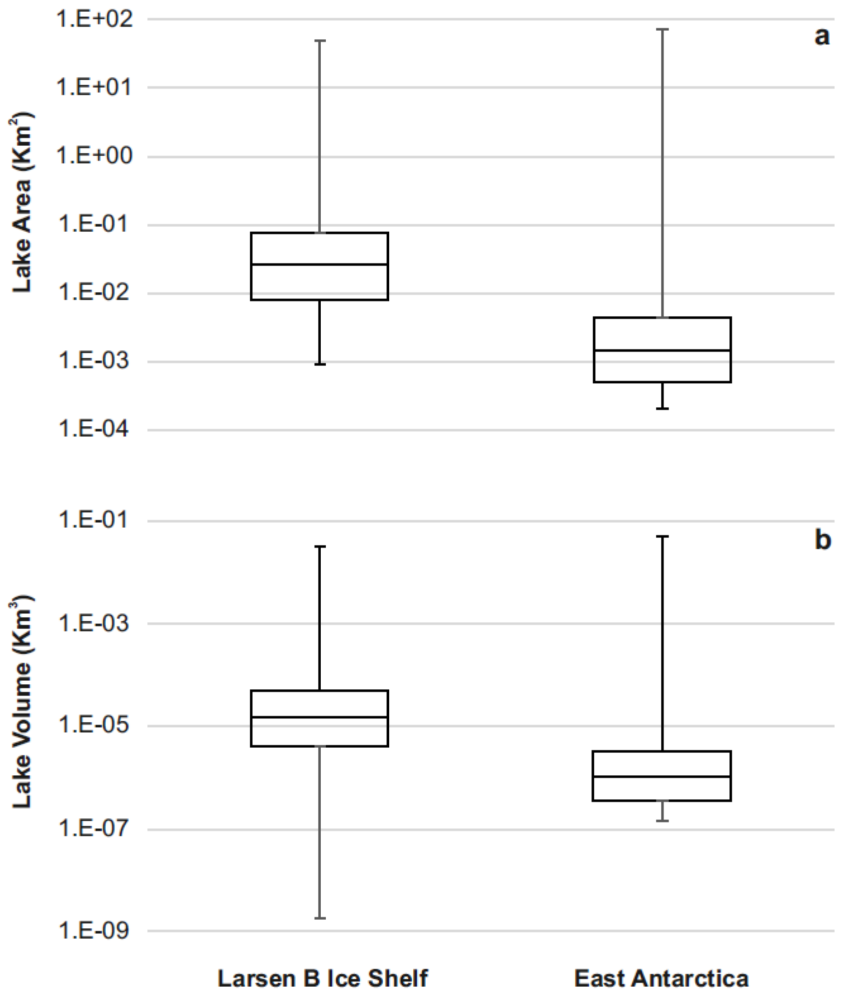


**Supplementary Figure 8: Box-and-whisker plots showing a comparison of SGL areas (a) and volumes (b) for the pre-collapse Larsen B ice shelf[56] and our dataset from East Antarctica.** The whiskers show the min-max range and the boxes display the interquartile range, with the median as the horizontal bar.
